# Elderberry (*Sambucus nigra* L.): an ethnopharmacological, phytochemical and biological review for a prospective nutraceutical plant

**DOI:** 10.1007/s10787-025-02017-0

**Published:** 2026-02-10

**Authors:** Asmaa M. Khalil, Rania M. Kamal, Riham A. El-Shiekh, Ahmed H. Elbanna, Sherif A. Hamdy

**Affiliations:** 1 Faculty of Pharmacy, Pharmacognosy Department, Cairo University, Cairo, 11562 Egypt; 2 Department of Pharmaceutical Sciences, College of Pharmacy, Gulf Medical University, 4184 Ajman, United Arab Emirates

**Keywords:** Elderberry, *Sambucus nigra* L, Traditional uses, Bioactive metabolites, Extracts optimization, Nutraceuticals

## Abstract

**Background:**

Elderberry (*Sambucus nigra* L.) has been traditionally implemented in diverse preparations such as herbal teas, syrups or juices as remedies for respiratory, febrile and other health conditions. Phytochemical and chromatographic analyses of different organs mapped their metabolite profiles and allowed identification, and sometimes isolation, of their main bioactive compounds.

**Aim of the study:**

Inspired by the rich and effective literature of *S. nigra*, this review article aims to summarize and highlight its reported biological (traditional and research-based) and chemical profiles.

**Methods:**

The Keywords used in the search included biological activities, pharmacological reports, phytochemistry, isolated compounds, taxonomy, botanical data, single or combination; traditional, traditionally, ethnopharmacology, folk uses, toxicity, LD50, interactions, side effects, clinical studies, elderberry, elder, *Sambucus nigra.* Using different bibliographic databases, Google Scholar, PubMed, Web of Science, Springer Link, and Science Direct with no specific limits.

**Results:**

In this context, elderberry is deemed a rich source for a myriad of bioactive compounds, mainly phenolics, and was proven to exhibit a variety of health benefits, including antioxidant, anti-inflammatory, anticancer, anti-influenza, antimicrobial, antidiabetic, cardioprotective, and neuroprotective properties. This review also covers different analysis approaches applied for *S. nigra* characterization in addition to literature studies attempted to optimize its extract(s) preparation process in terms of different extraction solvents, temperatures or methodologies to enrich prepared extracts in beneficial and bioactive metabolites.

**Conclusion:**

Overall, elderberry holds substantial potential as a rich dietary source of bioactive metabolites. Future research into its application in functional foods and nutraceuticals may provide innovative strategies for the prevention and management of various chronic diseases.

**Clinical Trial Number in the manuscript:**

Not applicable.

**Graphical abstract:**

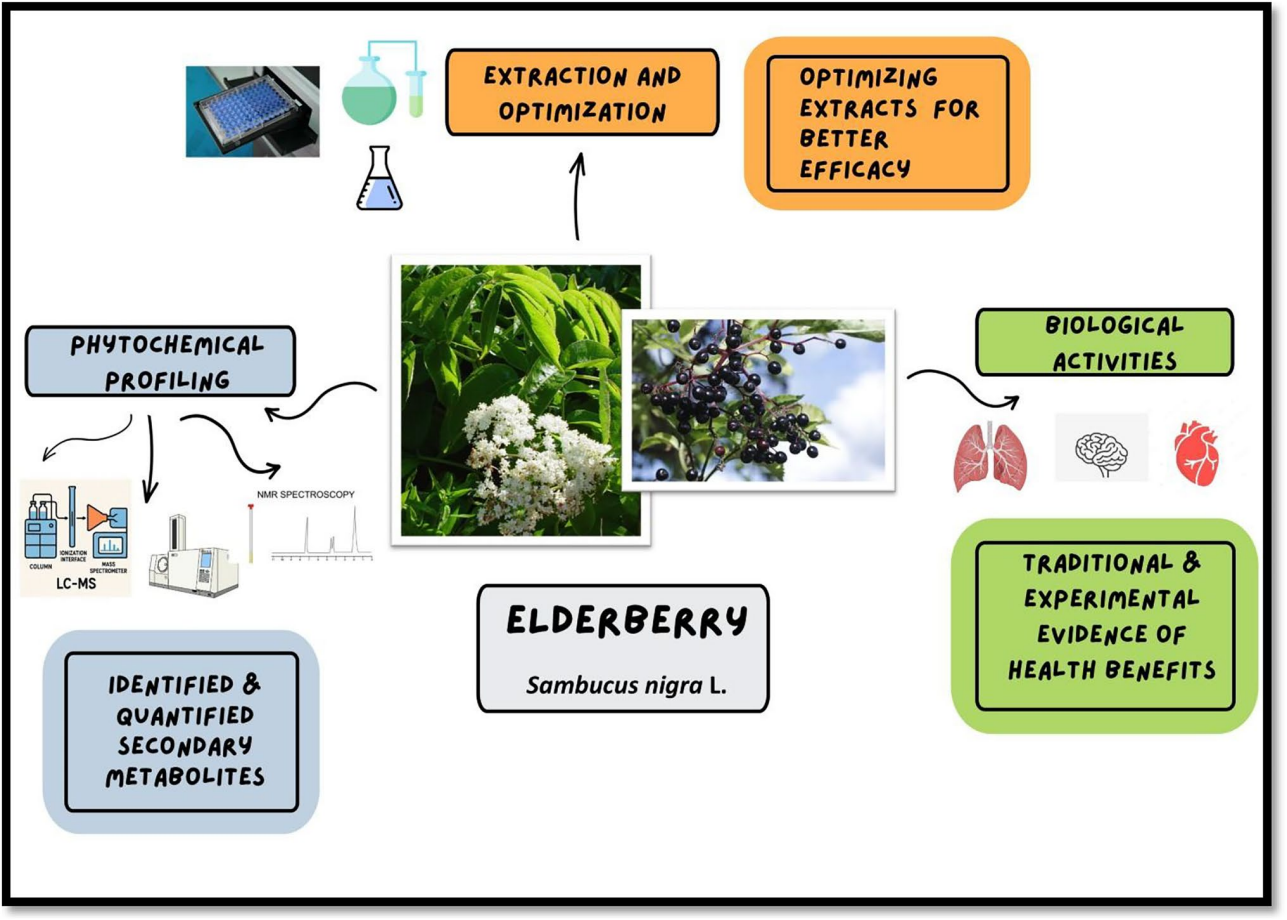

## Introduction

*Sambucus nigra* L. (Viburnaceae family), often known as European elder or black elder or elderberry, is a medicinal shrub or small tree found in Europe, Africa, Asia, and North America k. The plant’s flowers bloom from May to June, and fruiting begins in July. The fruits fully ripen between late August and early September (Atkinson and Atkinson 2002). For centuries, elderberries have been used for diverse medical purposes. According to the British Pharmacopoeia 1788, elderberry syrup, processed from ripe elderberry fruits, is effective against coughs, colds, and constipation. Since 1887, a decoction of dried elderberries has been used as a laxative in Germany, while the tea prepared from fresh elderberries has been used for the same purpose in Ukraine, Poland, Czechia, and Slovakia (EMA/HMPC 2018). The white flowers of *S. nigra* are also commonly utilized in folk medicine such as in Albania, Algeria, Italy, and Spain, where they have been consumed internally to cure bronchial infections, colds, and stomachaches, as well as been used as an antipyretic, diuretic, digestive, and antirheumatic (Motti et al. 2022). In this context, the dried flowers, or Sambuci flos, have been included in pharmacopoeias, and their therapeutic properties have been reported in several monographs. According to the World Health Organization (WHO) Monographs on Selected Medicinal Plants, elder flowers are diaphoretic, expectorant, and used for symptomatic treatment of common cold (2022). A similar report was also described in the European Union’s herbal monograph on elderberry flowers indicating that decoctions, infusions, liquid extracts, and tinctures of the flowers have long been used to alleviate early symptoms of common cold (EMA/HMPC 2018). Additionally, *S. nigra* was used in Turkish traditional medicine to treat wounds, rheumatic discomfort, common cold, and high fever (Süntar et al. 2010).

The high commercial value of *S. nigra* is mainly linked to its high phenolic content (Silva et al. 2017). Research-based studies attributed many of elderberry’s biological activities to its high phenolic content, including antioxidant (Dawidowicz et al. 2006), antiflu (Torabian et al. 2019), anti-obesity (Ulusoy et al. 2024), anti-inflammatory (Ho et al. 2017b), and antidiabetic (Studzińska-Sroka et al. 2024a) effects. Rutin (quercetin-3-*O*-rutinoside) is the most common flavonoid found throughout the plant, while other flavonoids found in the flowers, fruits, and leaves include quercetin, quercetin-3-*O*-glucoside (isoquercitrin), kaempferol-3-*O*-rutinoside, kaempferol-3-*O*-glucoside (astragaline), and quercetin-3-*O*-galactoside (hyperoside). In terms of phenolic acids, the plant is rich in chlorogenic, neochlorogenic, and cryptochlorogenic acids (Ferreira et al. 2022b; Młynarczyk et al. 2018).

Here in, we focused our research review on elderberry as a plant that has been underexplored and underutilized in the food and pharmaceutical industries. The review covers the reported health benefits of elderberry (traditional and experimental) in addition to its investigated phytochemical profiles in terms of isolated, identified, characterized, or quantified main secondary metabolites, and the analytical methods used for these studies. The review also summarizes the efforts exhibited to optimize different elderberry extracts/preparations in the scene of higher contents of desired metabolites and/or minimal contents of undesired ones (if any) (Domínguez et al. 2020). This optimization is crucial for better bioactivity profiles, which is a major concern to phytochemists, nutritionists, the health care sector and eventually consumers.

## Botanical profile and taxonomy

(Charlebois et al. 2010; https://mainenaturalhistory.org/product/free-mini-guide-black-elderberry-vs-red-elderberry/; https://www.istockphoto.com/photos/sambucus-nigra).

### Morphology

The elderberry plant forms shrubs and small trees in the family Adoxaceae. These plants are primarily native to temperate and subtropical regions across both hemispheres. They are characterized by their bushy appearance, with numerous straight canes emerging from the base, reaching heights of up to 9 m (30 feet) and occasionally up to 10 m (33 feet). It can live for 25 to 35 years under optimal conditions.

**Fig. 1:**
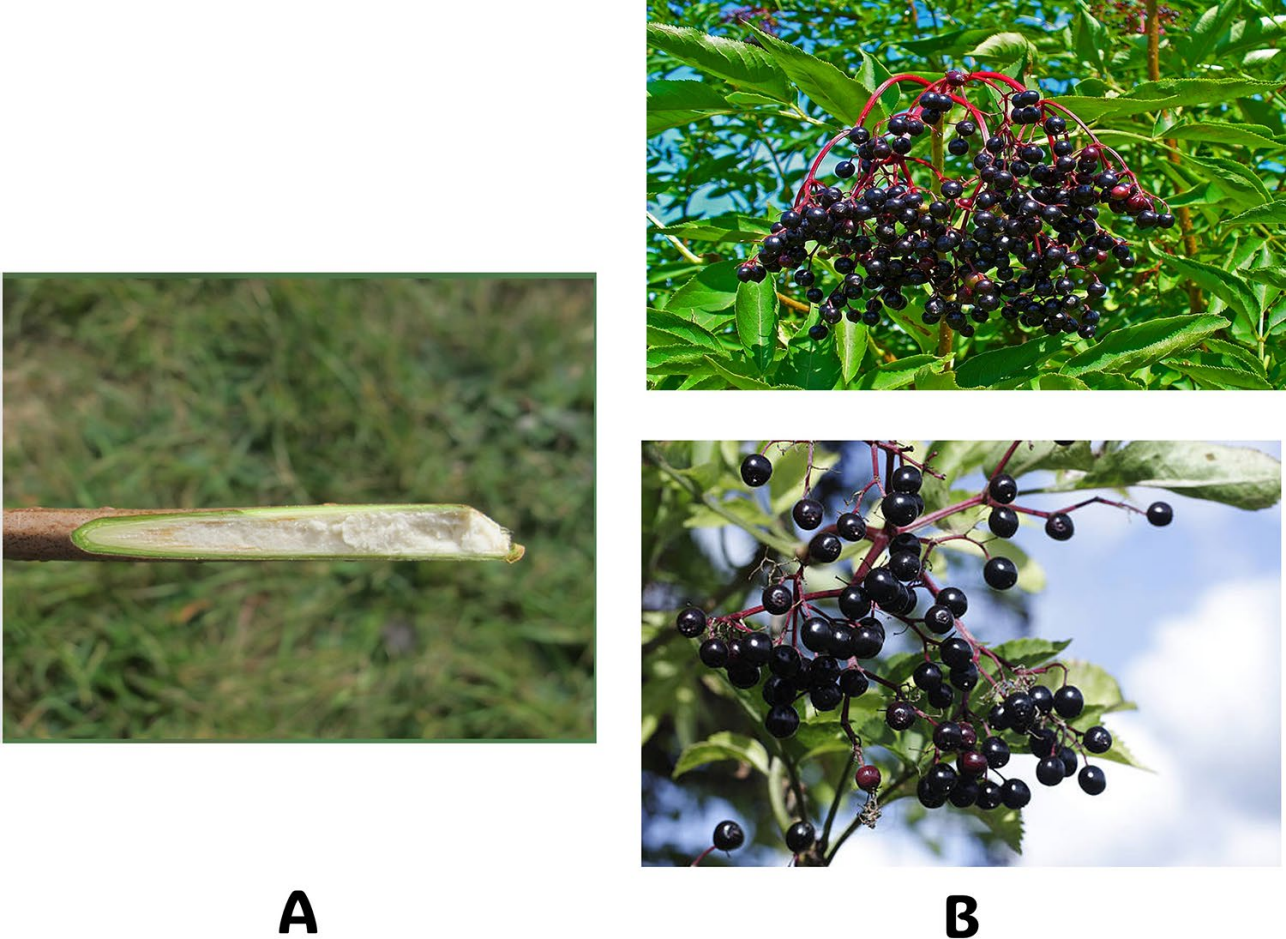
Elderberry **A** Canes and Bark, **B** Fruits (The copyright of the images belongs to the following websites: https://mainenaturalhistory.org/product/free-mini-guide-black-elderberry-vs-red-elderberry/; https://www.istockphoto.com/photos/sambucus-nigra)

Canes and Bark: The canes are weakly lignified and have a central white pith (Fig. 1A), making them somewhat brittle. The bark is typically light brown, yellowish, or grayish, featuring prominent lenticels and a deeply furrowed texture.

**Fig. 2:**
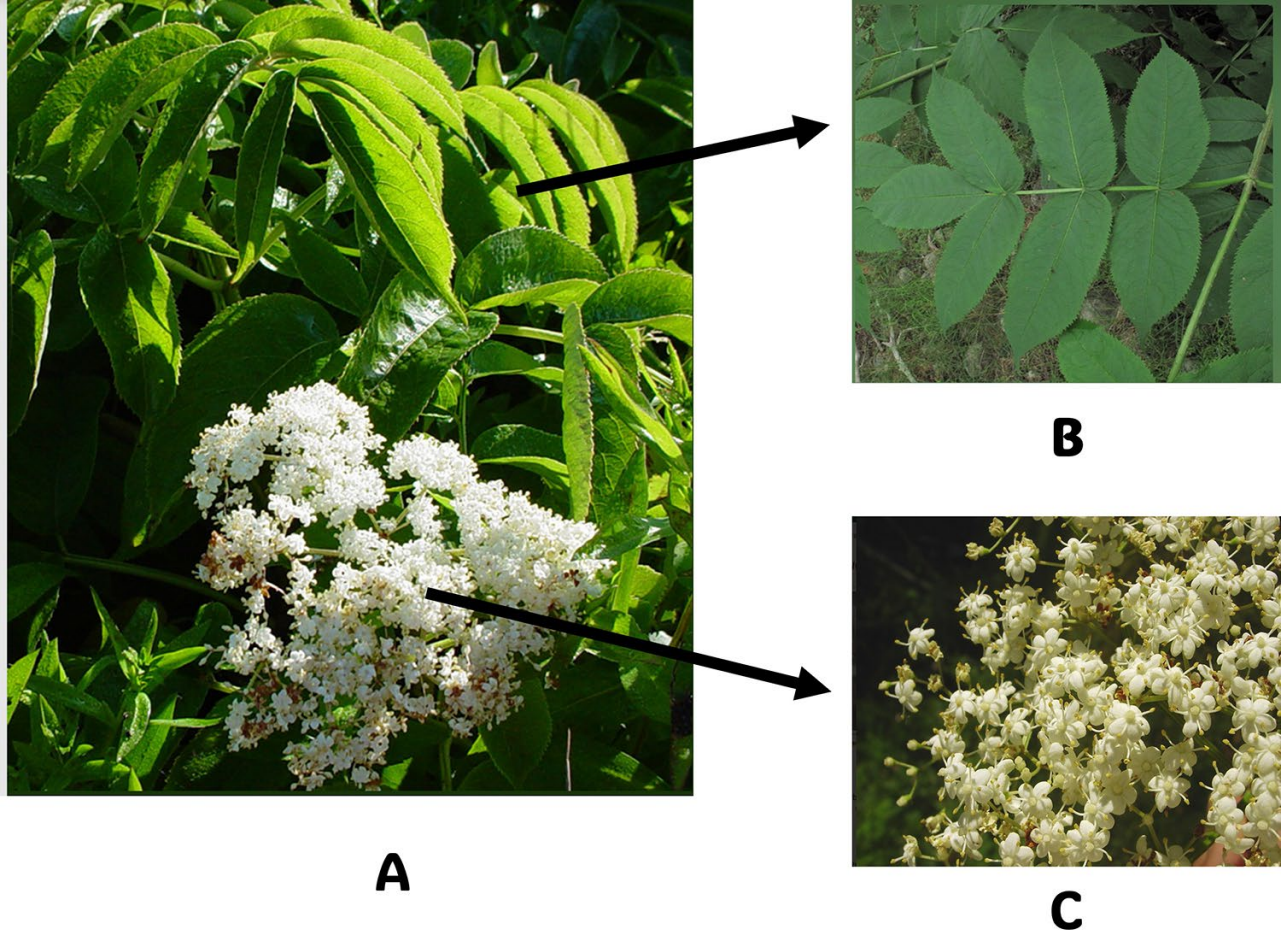
Elderberry **A**- Whole branch, **B**- Leaves, and **C**- Inflorescence (The copyright of the images belongs to the following websites: https://mainenaturalhistory.org/product/free-mini-guide-black-elderberry-vs-red-elderberry/; https://www.istockphoto.com/photos/sambucus-nigra)

Leaves: The leaves are opposite, stipulate, and odd-pinnately compound, consisting of 5 to 11 leaflets, most commonly 5 to 7. Leaflets range from 3 to 9 cm in length and exhibit a serrated margin. They are bright green to medium green on the upper side and hairy on the underside, particularly along the veins. The petiole can reach a length of 3 to 4 cm (Fig. 2A and B).

Inflorescence and Flowers: Elderberry produces flat-topped clusters of small white or cream-colored flowers in late spring. These flowers have a pleasant yet slightly rancid odor and are followed by berry-like drupes that ripen from green to red and finally black with a glossy appearance over 6 to 8 weeks from July to September (Fig. 2A and C).

Fruits: The fruits are spherical berries approximately 5–6 mm wide at maturity, containing several nutlets. They are rich in anthocyanins, contributing to their intense blue-purple coloration (Fig. 1B).

### Taxonomical data

**Table:** 

English names:	Black elderberry, Bour tree, Bore tree, Elder, Common elder, European elder
Kingdom:	Plantae
Order:	Dipsacales
Family:	Adoxaceae
Genus:	*Sambucus*
Species:	*Sambucus nigra* L

## Phytochemistry

### Secondary metabolites isolated from *Sambucus nigra*

**Fig. 3:**
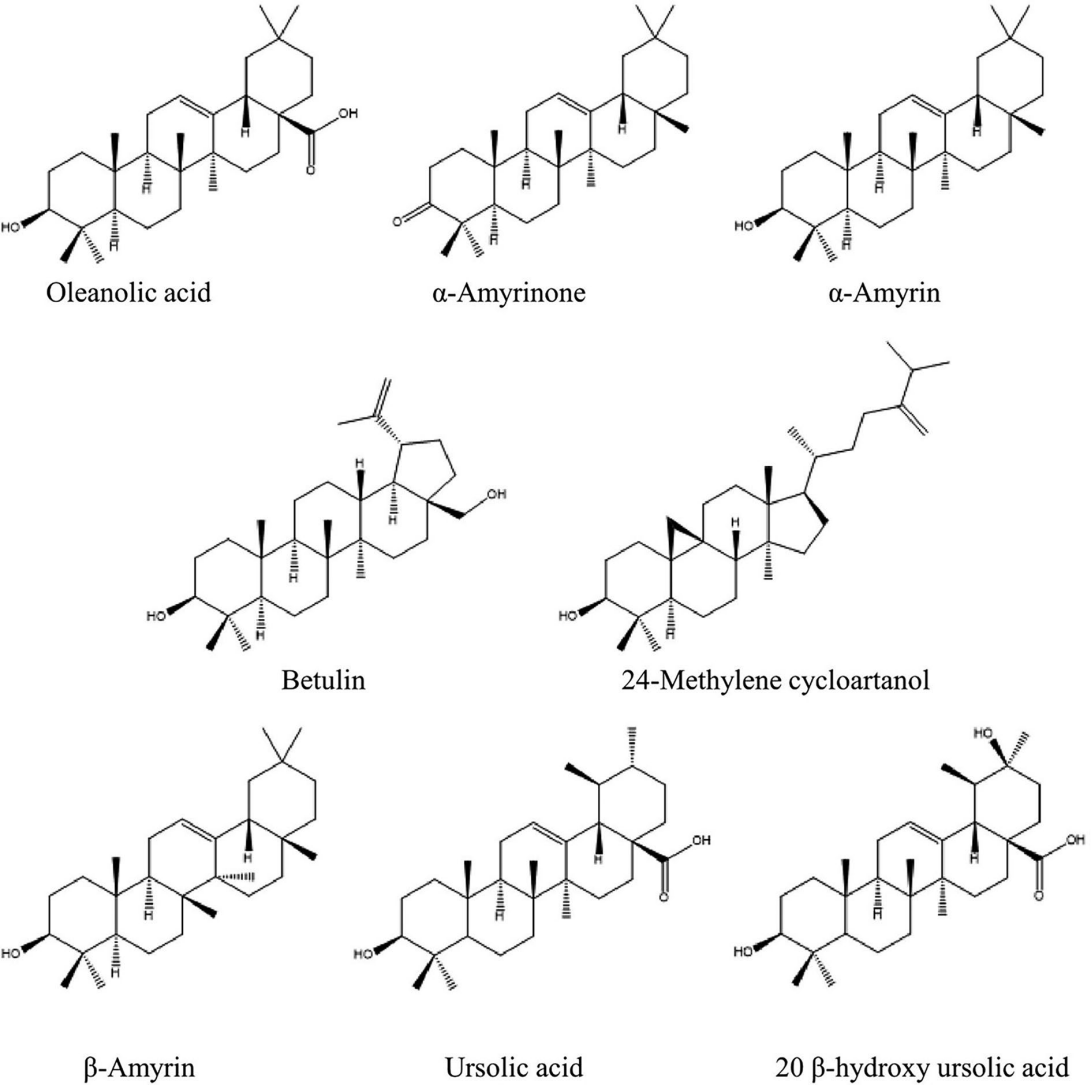
Triterpenoids isolated from *Sambucus nigra* (Elderberry)

**Fig. 4:**
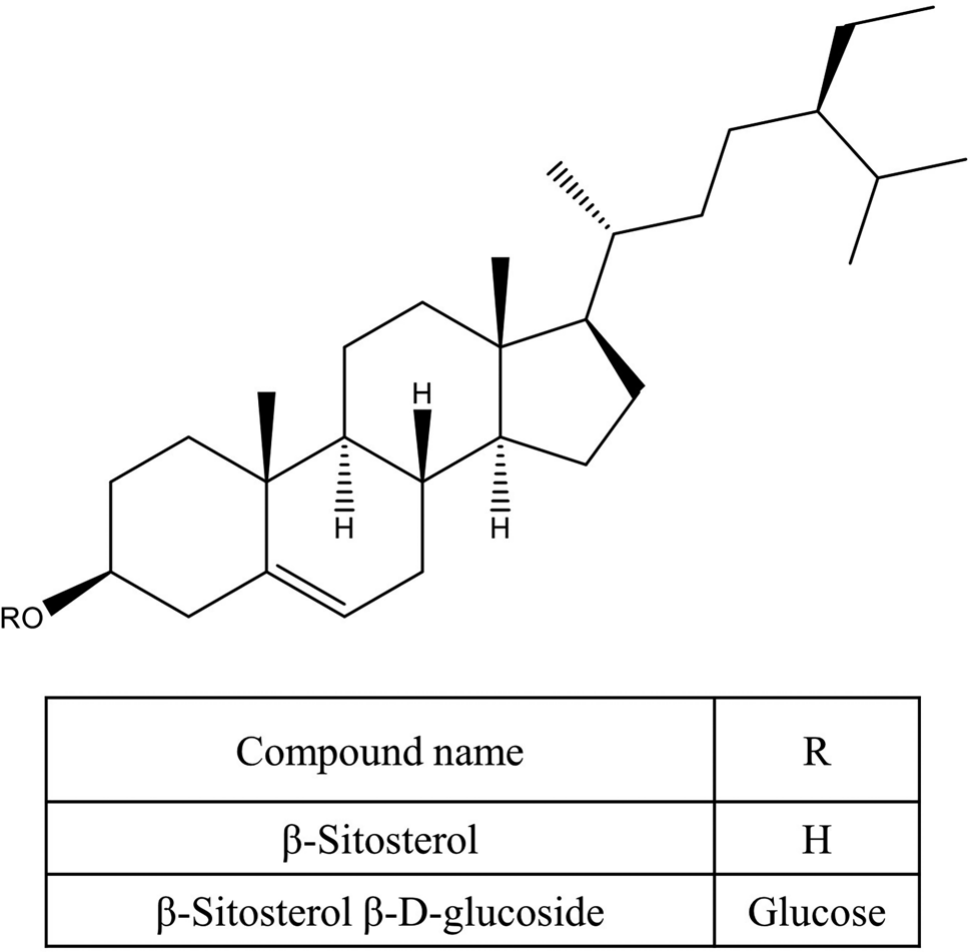
Sterols isolated from *Sambucus nigra* (Elderberry)

**Fig. 5:**
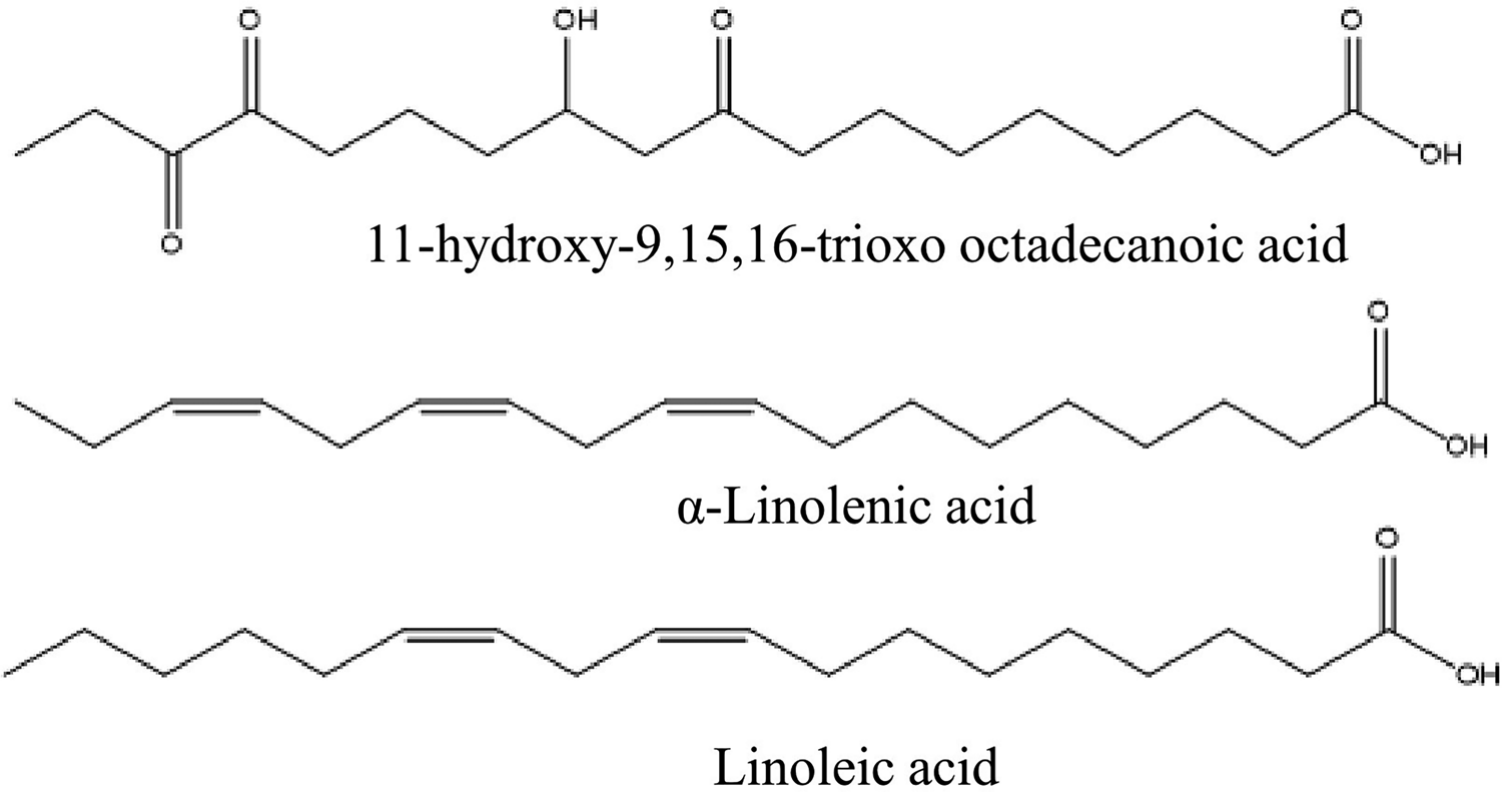
Fatty acids isolated from *Sambucus nigra* (Elderberry)

**Fig. 6:**
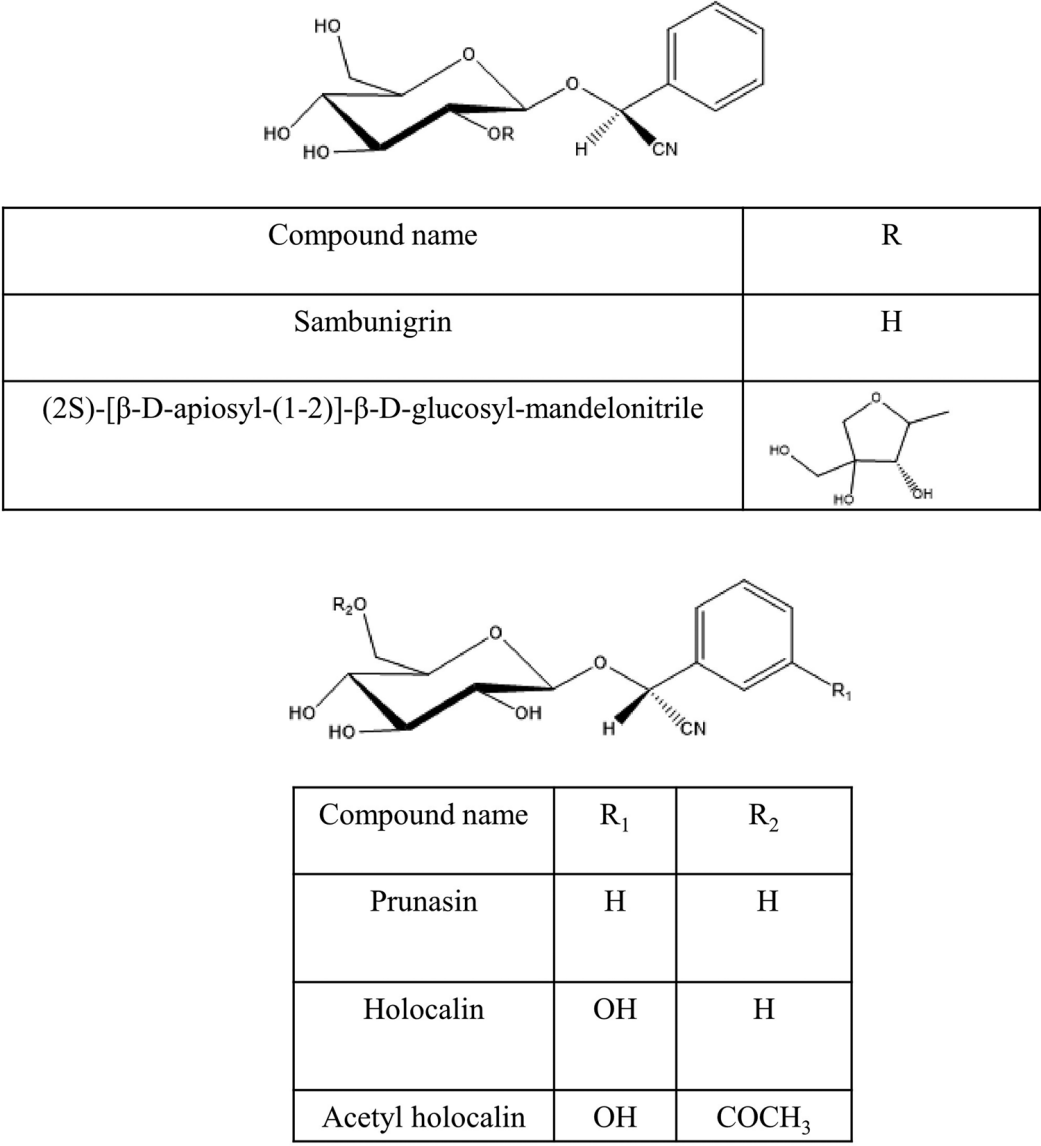
Cyanogenic glycosides isolated from *Sambucus nigra* (Elderberry)

**Fig. 7:**
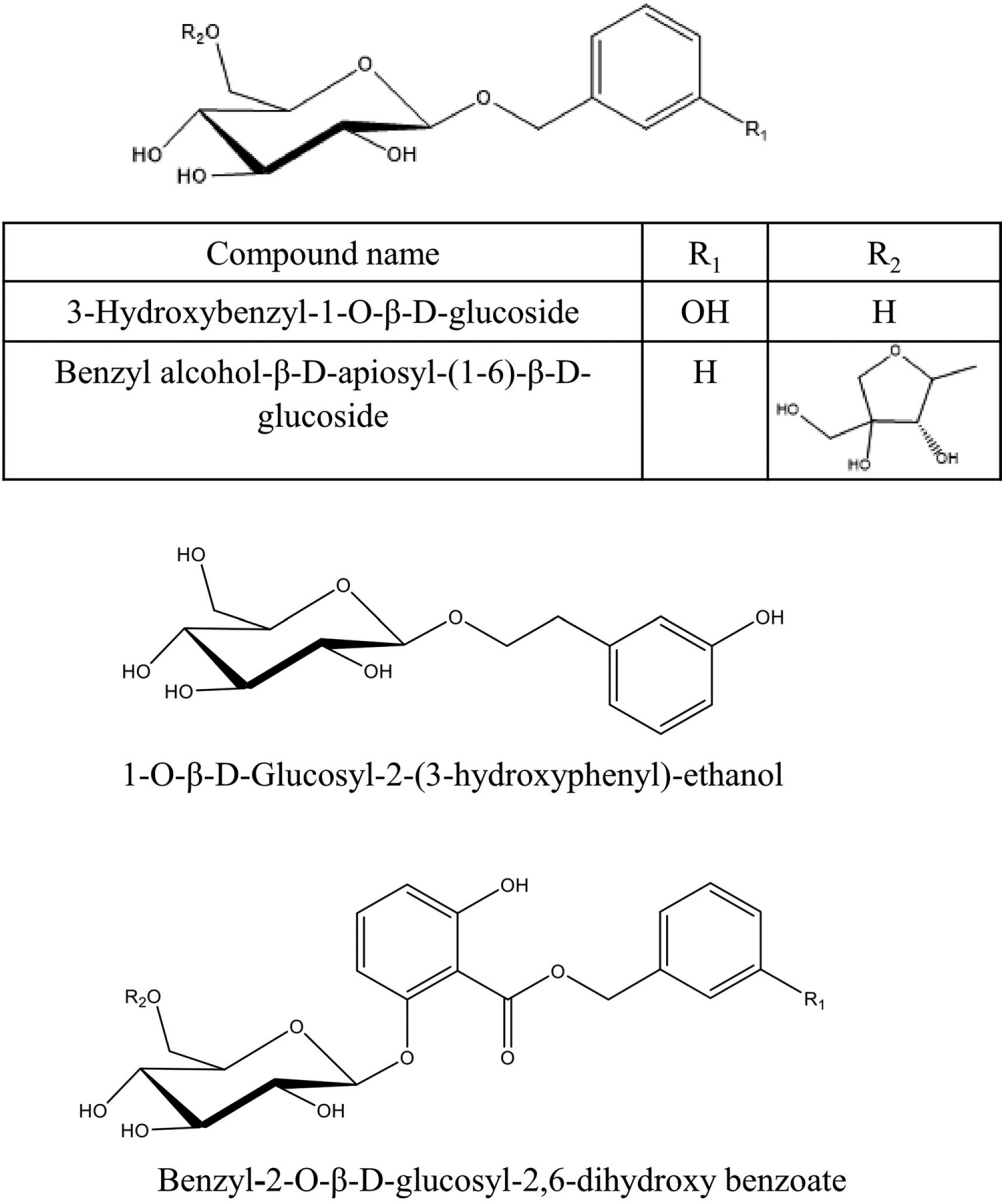
Phenolic glycosides isolated from *Sambucus nigra* (Elderberry)

**Fig. 8:**
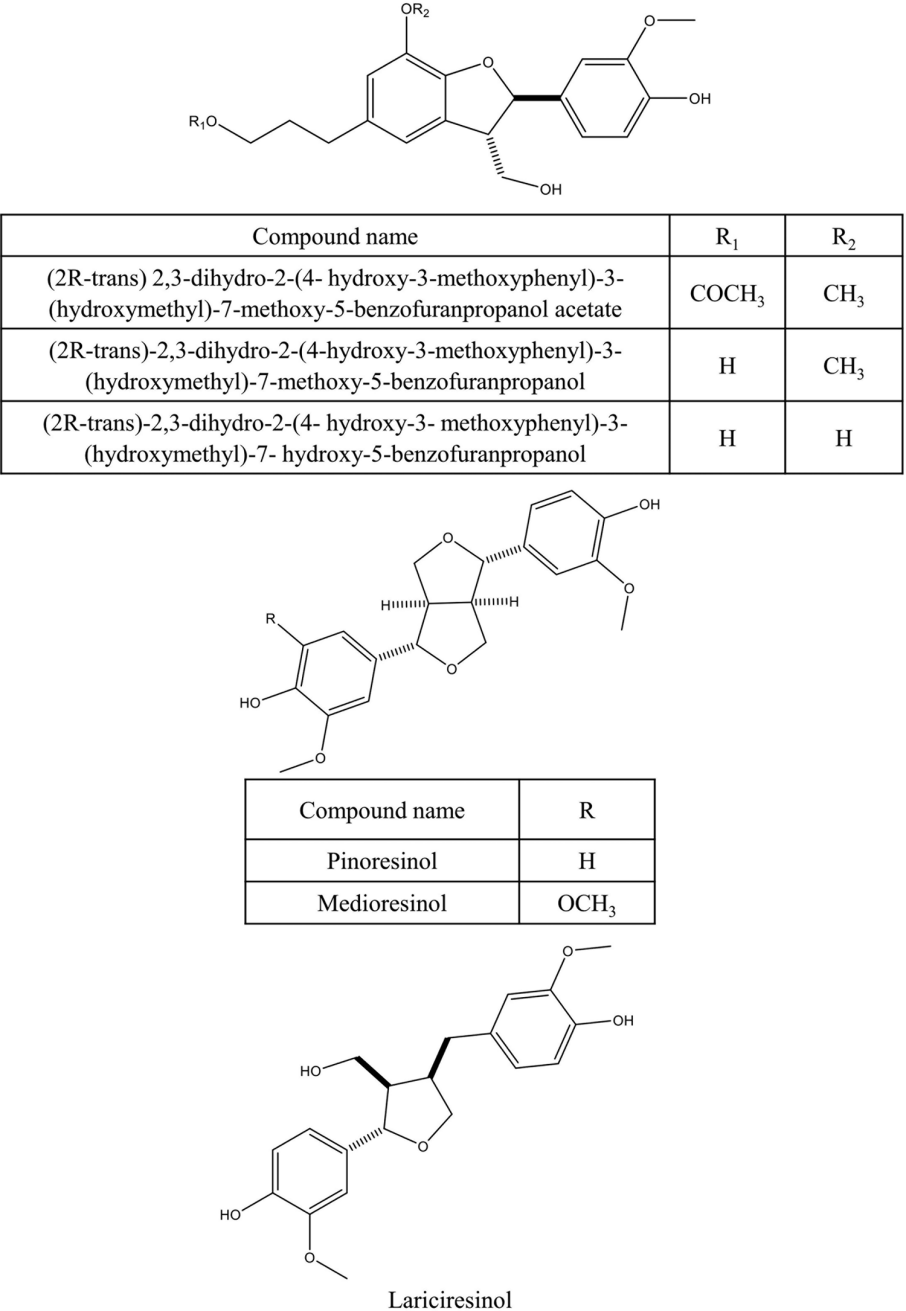
Lignans isolated from *Sambucus nigra* (Elderberry)

**Fig. 9:**
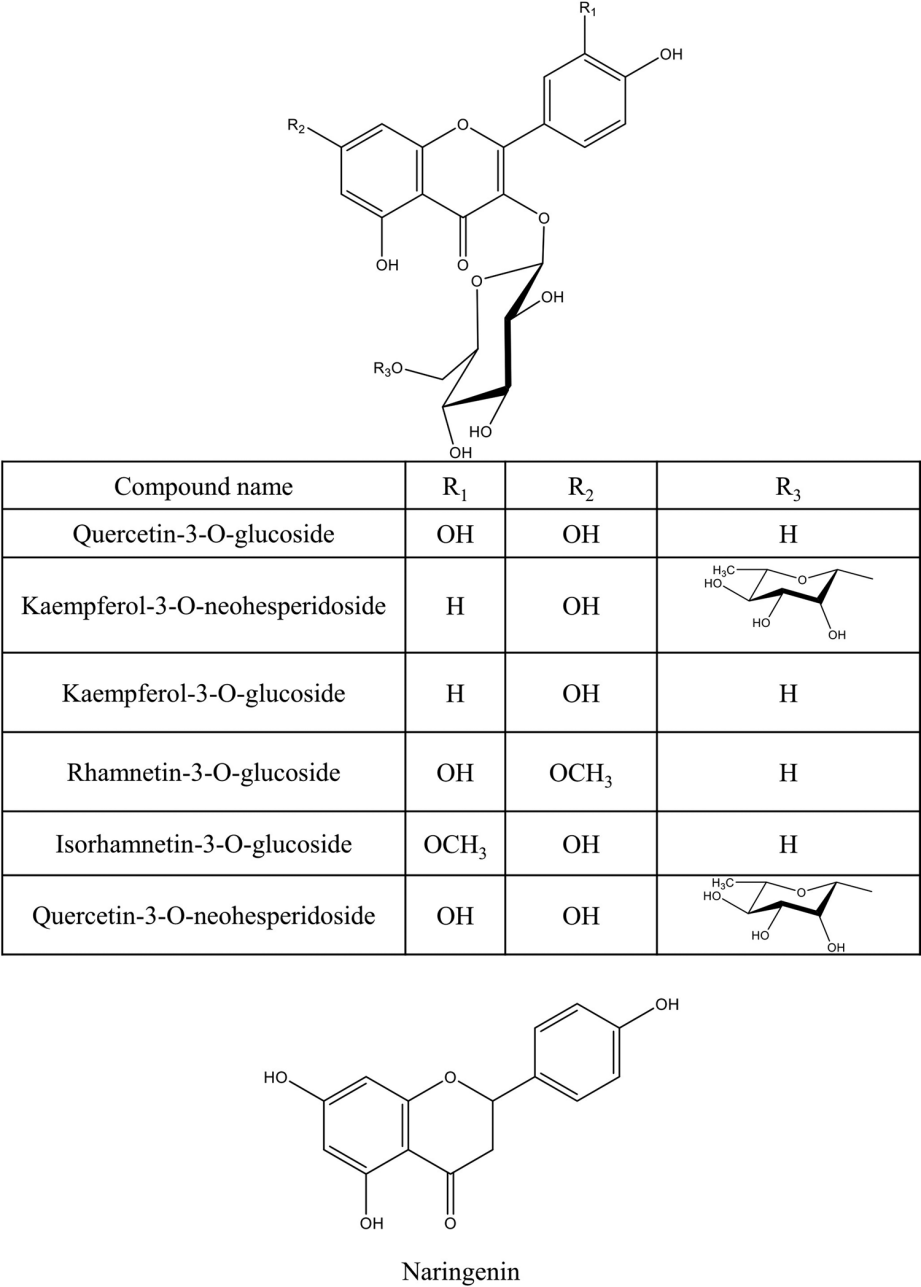
Flavonoids isolated from *Sambucus nigra* (Elderberry)

**Fig. 10:**
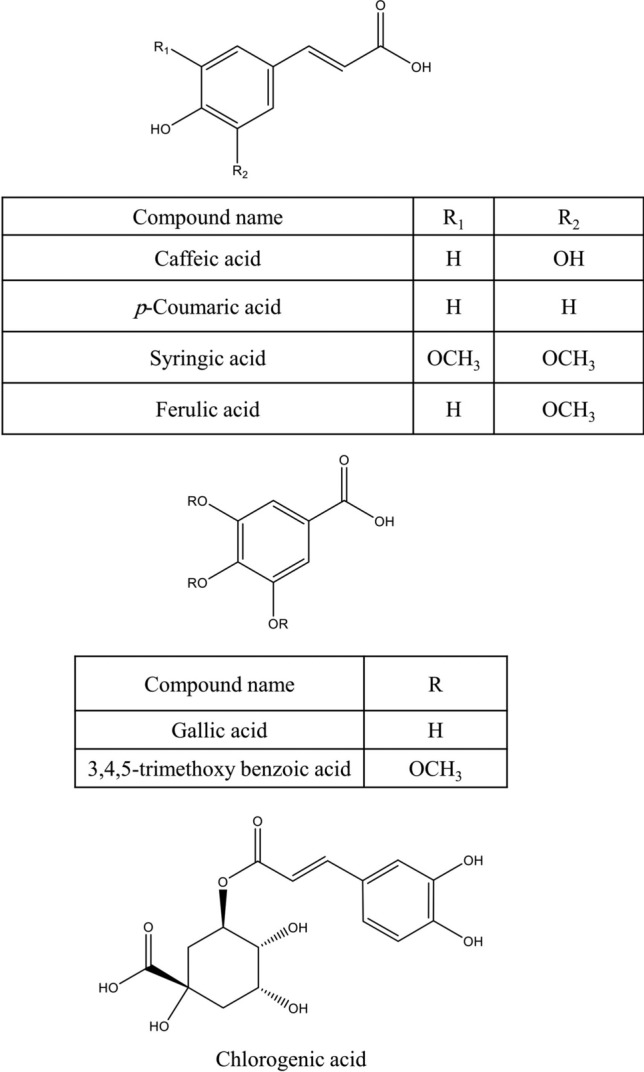
Phenolic acids isolated from *Sambucus nigra* (Elderberry)

Various secondary metabolites were isolated from different *S. nigra* organs *viz.* bark, seeds, flowers, leaves, and fruits. These compounds belong to a wide range of metabolic classes, including triterpenoids, sterols, fatty acids, cyanogenic glycosides, phenolic glycosides, lignans, flavonoids, and phenolic acids. Compounds isolated and reported from *S. nigra* are summarized in Table 1, along with methods of their isolation, structure elucidation, studied biological activities, and organs from which they were isolated, whereas their structures are presented in Figs. 3, 4, 5, 6, 7, 8, 9, 10.

**Table 1:** Compounds isolated from *Sambucus nigra* (Elderberry)

Compound name	Class	Organ	Extract	Isolation method	Structure elucidation technique(s)	Studied biological activity	References
Oleanolic acid	Triterpenoids	Bark	Petroleum ether	Repeated column chromatography of the non-saponifiable fraction of petroleum extract on alumina column followed by solvent crystallization	Melting point and IR spectroscopy	–	Lawrie et al. (1964)
*α*-Amyrinone							
α-Amyrin							
Betulin							
*β*-Sitosterol	Sterol						
11-Hydroxy-9,15,16- trioxo octadecanoic acid	Fatty acid	Seeds	Oil	Column chromatography followed by TLC purification	Refractive index, I_2_ number, and IR spectroscopy	–	Gigienova and Umarov (1972)
24-Methylene cycloartanol	Triterpenoids	Flowers	Methanol	Repeated column chromatography on KSKG silica gel and solvent crystallization	Melting point, MS, H^1^ NMR, and IR spectroscopy	–	Makarova and Isaev (1997)
*α*-Amyrin							
*β*-Amyrin							
*β*-Sitosterol							
Ursolic acid							
20 *β*-Hydroxy ursolic acid							
*β*-Sitosterol *β*-D-glucoside	Sterol glucoside						
Sambunigrin	Cyanogenic glycosides	Leaves	Methanol	Repeated column chromatography on different stationary phases and preparative HPLC	UV absorption, EI-MS, and H^1^ NMR spectroscopy	Inhibitory effect on seed germination and radicle elongation	D'Abrosca et al. (2001)
Prunasin							
Holocalin							
Acetyl holocalin							
(2*S*)-[*β*-D-apiosyl-(1–2)]-*β*-D-glucosyl-mandelonitrile							
3-Hydroxybenzyl-1-*O*-*β*-D-glucoside	Phenolic glycosides					–	
1-*O*-*β*-D-Glucosyl-2-(3-hydroxyphenyl)-ethanol							
Benzyl alcohol-β-D-apiosyl-(1–6)-*β*-D-glucoside							
Benzyl-2-*O*-*β*-D-glucosyl-2,6-dihydroxy benzoate							
(2*R*-*trans*) 2,3-Dihydro-2-(4-hydroxy-3-methoxyphenyl)-3-(hydroxymethyl)-7-methoxy-5-benzofuranpropanol acetate	Neolignans					Stimulatory effect on seed germination and radicle elongation	
(2*R*-trans)-2,3-dihydro-2-(4-hydroxy-3-methoxyphenyl)-3- (hydroxymethyl)-7-methoxy-5-benzofuranpropanol							
(2*R*-*trans*)-2,3-dihydro-2-(4- hydroxy-3- methoxyphenyl)-3- (hydroxymethyl)-7- hydroxy-5-benzofuranpropanol							
Pinoresinol	Lignans						
Medioresinol							
Lariciresinol							
Quercetin-3-*O*-glucoside	Flavonol glycosides					–	
Kaempferol-3-*O*-neohesperidoside							
Kaempferol-3-*O*-glucoside							
Rhamnetin-3-*O*-glucoside							
Quercetin-3-*O*-neohesperidoside							
Caffeic acid	Phenolic acids	Bark	70% methanol	Successive column chromatography on polyamide and Sephadex LH-20 columns and preparative TLC	Melting point, negative ESI-TOF MS, and H^1^ NMR spectrometry	–	Turek and Cisowski (2007)
Gallic acid							
*p*-Coumaric acid							
3,4,5-Trimethoxy benzoic acid							
Chlorogenic acid							
Syringic acid							
Ferulic acid							
*α*-Linolenic acid	Fatty acids	Flowers	Methanol	Flash column chromatography on RP-18 silica gel column followed by semi-preparative HPLC	MS and HPLC against standards	PPARγ activation	Christensen et al. (2010)
Linoleic acid							
Naringenin	Flavanone						
Quercetin-3-*O*-glucoside	Flavonol glycosides	Leaves	Methanol	Successive chemical separation techniques of butanol fraction including precipitation, ion-exchange, silica gel, and Sephadex column chromatography	H^1^, C^13^ NMR, and FAB-MS	Antiulcer	Yesilada et al. (2014)
Isorhamnetin-3-*O*-glucoside

### Chromatographic analysis of *Sambucus nigra* extracts

Different organs of *S. nigra* were extensively investigated using different liquid and gas chromatographic techniques for metabolites profiling and identification. The applied chromatographic techniques, together with the resulting identified metabolites, are briefly discussed in the upcoming sections.

### LC/MS analysis of *Sambucus nigra* extracts

Liquid Chromatography coupled to Mass Spectrometry (LC/MS) investigation of various extracts prepared from different *S. nigra* organs, in addition to some processed products prepared from flowers or berries (e.g. syrup, juice, tea, liqueur, and spread) led to the identification of a myriad of metabolites belonging to numerous phytochemical classes including phenolic acids, flavonoids, anthocyanins, lignans, catechins, coumarins, iridoids, fatty acids, organic acids, triterpenoids, and cyanogenic glycosides. The identified metabolites are listed in Table 2 along with the LC/MS technique utilized for their identification, and specifications of the studied extract(s), processed product(s) or organ(s).

**Table 2:** Metabolites identified in different *Sambucus nigra* (Elderberry) extracts through LC/MS techniques

Name	Organ	Extract	Identification technique	References
*Phenolic acids*				
5-Caffeoyl quinic acid (neochlorogenic acid)	Flowers	Methanol	HPLC–DAD-MS	Christensen et al. (2008)
	Flowers	Dichloromethane and methanol	LC-PDA-MS	Bhattacharya et al. (2013)
	Berries	Juice, liqueur, tea, and spread	HPLC–DAD-MS^n^	Senica et al. (2016)
	Leaves, flowers, and berries	70% methanol	HPLC–DAD-MS^n^	Senica et al. (2019)
	Flowers	50% ethanol and water	LC–MS/MS	Milena et al. (2019)
	Flowers	Aqueous	HPLC–PDA-MS^n^	Ferreira-Santos et al. (2021)
	Flowers	Aqueous infusion, 80% methanol, and 80% ethanol	LC-DAD-ESI–MS/MS	Uzlasir et al. (2021)
	Flowers and leaves	Methanol	LC-DAD-ESI-MS^n^	Qazimi et al. (2024)
*p*-Coumaroyl quinic acid Di caffeoyl quinic acid	Flowers	Methanol	HPLC–DAD-MS	Christensen et al. (2008)
	Berries	Juice, liqueur, and spread	HPLC–DAD-MS^n^	Senica et al. (2016)
	Leaves, flowers, and berries	70% methanol	HPLC–DAD-MS^n^	Senica et al. (2019)
	Flowers	Aqueous	HPLC–PDA-MS^n^	Ferreira-Santos et al. (2021)
	Flowers	Aqueous infusion, 80% methanol, and 80% ethanol	LC-DAD-ESI–MS/MS	Uzlasir et al. (2021)
	Berries and flowers	Acidified methanol (1% formic acid in methanol)	UHPLC-PDA-MS	Avula et al. (2022)
	Flowers and leaves	Methanol	LC-DAD-ESI-MS^n^	Qazimi et al. (2024)
*p*-Coumaric acid	Flowers	Dichloromethane and methanol	LC-PDA-MS	Bhattacharya et al. (2013)
	Berries	Juice, liqueur, tea, and spread	HPLC–DAD-MS^n^	Senica et al. (2016)
	Berries	70% methanol	HPLC–DAD-MS^n^	Senica et al. (2019)
	Flowers	50% ethanol and water	LC–MS/MS	Milena et al. (2019)
Ferulic acid	Flowers	Dichloromethane and methanol	LC-PDA-MS	Bhattacharya et al. (2013)
		50% ethanol and water	LC–MS/MS	Milena et al. (2019)
Caffeic acid	Flowers	Dichloromethane and methanol	LC-PDA-MS	Bhattacharya et al. (2013)
	Flowers	50% ethanol and water	LC–MS/MS	Milena et al. (2019)
	Berries and flowers	Acidified methanol (1% formic acid in methanol)	UHPLC-PDA-MS	Avula et al. (2022)
3-Caffeoyl quinic acid (chlorogenic acid) 4-Caffeoyl quinic acid (cryptochlorogenic acid)	Berries	Juice, liqueur, tea, and spread	HPLC–DAD-MS^n^	Senica et al. (2016)
	Leaves, flowers, and berries	70% methanol	HPLC–DAD-MS^n^	Senica et al. (2019)
	Flowers	Aqueous	HPLC–PDA-MS^n^	Ferreira-Santos et al. (2021)
	Flowers	Aqueous infusion, 80% methanol, and 80% ethanol	LC-DAD-ESI–MS/MS	Uzlasir et al. (2021)
	Berries and flowers	Acidified methanol (1% formic acid in methanol)	UHPLC-PDA-MS	Avula et al. (2022)
	Flowers and leaves	Methanol	LC-DAD-ESI-MS^n^	Qazimi et al. (2024)
*p*-Coumaric acid glucoside	Berries	Juice, liqueur, tea, and spread	HPLC–DAD-MS^n^	Senica et al. (2016)
	Leaves, flowers, and berries	70% methanol	HPLC–DAD-MS^n^	Senica et al. (2019)
	Berries and flowers	Acidified methanol (1% formic acid in methanol)	UHPLC-PDA-MS	Avula et al. (2022)
Protocatechuic acid Vanillic acid Gallic acid Ellagic acid	Flowers	50% ethanol and water	LC–MS/MS	Milena et al. (2019)
Caffeic acid hexoside	Leaves, flowers, and berries	70% methanol	HPLC–DAD-MS^n^	Senica et al. (2019)
Feruloyl quinic acid	Leaves, flowers, and berries	70% methanol	HPLC–DAD-MS^n^	Senica et al. (2019)
	Flowers	Aqueous	HPLC–PDA-MS^n^	Ferreira-Santos et al. (2021)
	Flowers	Aqueous infusion, 80% methanol, and 80% ethanol	LC-DAD-ESI–MS/MS	Uzlasir et al. (2021)
	Berries and flowers	Acidified methanol (1% formic acid in methanol)	UHPLC-PDA-MS	Avula et al. (2022)
Glucocaffeic acid	Berries and flowers	Acidified methanol (1% formic acid in methanol)	UHPLC-PDA-MS	Avula et al. (2022)
Gentisic acid *p*-Hydroxybenzoic acid	Flowers	50% ethanol and water	LC–MS/MS	(Milena et al. (2019)
	Berries and flowers	Acidified methanol (1% formic acid in methanol)	UHPLC-PDA-MS	Avula et al. (2022)
*p-*Coumaroyl caffeoyl quinic acid	Leaves and flowers	70% methanol	HPLC–DAD-MS^n^	Senica et al. (2019)
	Flowers	Aqueous	HPLC–PDA-MS^n^	Ferreira-Santos et al. (2021)
	Berries and flowers	Acidified methanol (1% formic acid in methanol)	UHPLC-PDA-MS	Avula et al. (2022)
	Flowers and leaves	Methanol	LC-DAD-ESI-MS^n^	Qazimi et al. (2024)
Caftaric acid	Flowers and leaves	Methanol	LC-DAD-ESI-MS^n^	Qazimi et al. (2024)
*Flavonoids*				
Quercetin-3-rutinoside Kaempferol-3-rutinoside Isorhamnetin-3-rutinoside	Flowers	Methanol	HPLC–DAD-MS	Christensen et al. (2008)
	Flowers	Dichloromethane and methanol	LC-PDA-MS	Bhattacharya et al. (2013)
	Berries	Juice, liqueur, tea, and spread	HPLC–DAD-MS^n^	Senica et al. (2016)
	Leaves, flowers, and berries	70% methanol	HPLC–DAD-MS^n^	Senica et al. (2019)
	Flowers	Aqueous	HPLC–PDA-MS^n^	Ferreira-Santos et al. (2021)
	Flowers	Aqueous infusion, 80% methanol, and 80% ethanol	LC-DAD-ESI–MS/MS	Uzlasir et al. (2021)
	Berries and flowers	Acidified methanol (1% formic acid in methanol)	UHPLC-PDA-MS	Avula et al. (2022)
	Flowers and leaves	Methanol	LC-DAD-ESI-MS^n^	Qazimi et al. (2024)
Naringenin	Flowers	Methanol	LC–PAD–MS	Christensen et al. (2010)
		Dichloromethane and methanol	LC-PDA-MS	Bhattacharya et al. (2013)
		50% ethanol and water	LC–MS/MS	Milena et al. (2019)
		Syrup	UPLC–PDA–MS/MS	Matłok et al. (2021)
		Acidified methanol (1% formic acid in methanol)	UHPLC-PDA-MS	Avula et al. (2022)
	Flowers and leaves	Methanol	LC-DAD-ESI-MS^n^	Qazimi et al. (2024)
Quercetin-3-glucoside	Flowers	Dichloromethane and methanol	LC-PDA-MS	Bhattacharya et al. (2013)
	Berries	Juice, liqueur, tea, and spread	HPLC–DAD-MS^n^	Senica et al. (2016)
	Flowers	Aqueous infusion, 80% methanol, and 80% ethanol	LC-DAD-ESI–MS/MS	Uzlasir et al. (2021)
	Flowers and leaves	Methanol	LC-DAD-ESI-MS^n^	Qazimi et al. (2024)
Isorhamnetin-3-*O*-glucoside Quercetin-3-*O*-acetyl hexoside	Flowers	Dichloromethane and methanol	LC-PDA-MS	Bhattacharya et al. (2013)
	Leaves, flowers, and berries	70% methanol	HPLC–DAD-MS^n^	Senica et al. (2019)
Quercetin-hexoside pentoside	Berries	Juice, liqueur, tea, and spread	HPLC–DAD-MS^n^	Senica et al. (2016)
	Leaves, flowers, and berries	70% methanol	HPLC–DAD-MS^n^	Senica et al. (2019)
Naringenin hexoside	Berries	Liqueur and spread	HPLC–DAD-MS^n^	Senica et al. (2016)
		70% methanol	HPLC–DAD-MS^n^	Senica et al. (2019)
Morin	Flowers	50% ethanol and water	LC–MS/MS	Milena et al. (2019)
Isorhamnetin rhamnoside Isorhamnetin di glucoside Kaempferol-3-galactoside	Leaves, flowers, and berries	70% methanol	HPLC–DAD-MS^n^	Senica et al. (2019)
Quercetin-3-xyloside	Berries			
Quercetin-3-rhamnoside	Leaves			
Isorhamnetin Kaempferol-3-*O*-glucoside	Flowers	50% ethanol and water	LC–MS/MS	Milena et al. (2019)
	Leaves, flowers, and berries	70% methanol	HPLC–DAD-MS^n^	Senica et al. (2019)
	Berries and flowers	Acidified methanol (1% formic acid in methanol)	UHPLC-PDA-MS	Avula et al. (2022)
Isorhamnetin acetyl hexoside	Leaves, flowers, and berries	70% methanol	HPLC–DAD-MS^n^	Senica et al. (2019)
	Flowers	Aqueous	HPLC–PDA-MS^n^	Ferreira-Santos et al. (2021)
Kaempferol dihexoside	Flowers	70% methanol	HPLC–DAD-MS^n^	Senica et al. (2019)
		Aqueous infusion, 80% methanol, and 80% ethanol	LC-DAD-ESI–MS/MS	Uzlasir et al. (2021)
Quercetin trisaccharide	Flowers	Aqueous	HPLC–PDA-MS^n^	Ferreira-Santos et al. (2021)
Naringenin-5,7-*O*-di-glucoside Naringenin-7-*O*-rutinoside-5-*O*- pentoside Kaempferol-3-*O*-di-glucoside Kaempferol-3,7-*O*-di-glucosid Kaempferol-3-*O*-rhamnoside-7-*O*-pentoside Quercetin-3-*O*-rutinoside-7-*O*- glucoside Quercetin-3-*O*-rutinoside-7-*O*- pentoside Quercetin-3-*O*-rutinoside-7-*O*- rhamnoside Quercetin-3-*O*- glucoside-pentoside Quercetin-3-*O*-glucoside-7-*O*- glucuronide Quercetin-3-*O*-glucuronide Quercetin-7-methyl ether	Flowers	Syrup	UPLC–PDA–MS/MS	Matłok et al. (2021)
Quercetin-3-*O*-acetylglucoside	Flowers	Aqueous	HPLC–PDA-MS^n^	Ferreira-Santos et al. (2021)
	Flowers	Syrup	UPLC–PDA–MS/MS	Matłok et al. (2021)
	Berries and flowers	Acidified methanol (1% formic acid in methanol)	UHPLC-PDA-MS	Avula et al. (2022)
Quercetin dihexoside	Flowers	Aqueous	HPLC–PDA-MS^n^	Ferreira-Santos et al. (2021)
	Flowers	Aqueous infusion, 80% methanol, and 80% ethanol	LC-DAD-ESI–MS/MS	Uzlasir et al. (2021)
	Flowers and leaves	Methanol	LC-DAD-ESI-MS^n^	Qazimi et al. (2024)
Quercetin	Flowers	Syrup	UPLC–PDA–MS/MS	Matłok et al. (2021)
	Berries and flowers	Acidified methanol (1% formic acid in methanol)	UHPLC-PDA-MS	Avula et al. (2022)
Isorhamnetin-rutinoside-glucoside Kaempferol Quercetin-3-laminaribioside Isorhamnetin-3-laminaribioside	Flowers	Acidified methanol (1% formic acid in methanol)	UHPLC-PDA-MS	Avula et al. (2022)
Isorhamnetin-3-*O*-(acetyl galactoside) Quercetin 3-rhamninoside Quercetin di-glucoside Quercetin-3-arabinoglucoside Quercetin 3-*O*-malonylglucoside	Berries and flowers			
Caffeoyl kaempferol Quercetin coumaroyl rhamno-glucoside Quercetin coumaroyl rhamno-glucoside Quercetin malonyl diglucoside Kaempferol coumaroyl rhamno- glucoside Quercetin caffeoyl pentoside Isorhamnetin diglucoside Kaempferol coumaroyl glucoside Kaempherol-3-malonylglucosede Quercetin galloyl pentoside Isorhamnetin octylglucoside Acetyl-isoorientin Hydroxy trimethoxy flavonoid	Flowers and leaves	Methanol	LC-DAD-ESI-MS^n^	Qazimi et al. (2024)
*Anthocyanins*				
Cyanidin-3-*O*-glucoside Cyanidin-3-*O*-sambioside-5-*O*- glucoside Cyanidin-3-*O*-sambubioside Cyanidin-3,5-di-*O*-glucoside Cyanidin-3-*O*-rutinoside Pelargonidin-3-*O*-glucoside Pelargonidin-3-*O*-sambubioside	Berries	Juice, liqueur, tea, and spread	HPLC–DAD-MS^n^	Senica et al. (2016)
		70% methanol	HPLC–DAD-MS^n^	Senica et al. (2019)
		Acidified methanol (1% formic acid in methanol)	UHPLC-PDA-MS	Avula et al. (2022)
		Juice, liqueur, tea, and spread	HPLC–DAD-MS^n^	Senica et al. (2016)
		80% methanol	UPLC-qToF-ESI/MS–MS	Porras-Mija et al. (2020)
		Acidified methanol (1% formic acid in methanol)	UHPLC-PDA-MS	Avula et al. (2022)
*Coumarins*				
Esculetin	Flowers	50% ethanol and water	LC–MS/MS	Milena et al. (2019)
*Catechins*				
Catechin Epicatechin	Berries	Juice, liqueur, and spread	HPLC–DAD-MS^n^	Senica et al. (2016)
	Berries	70% methanol	HPLC–DAD-MS^n^	Senica et al. (2019)
	Flowers	50% ethanol and water	LC–MS/MS	Milena et al. (2019)
Procyanidin dimer	Berries	70% methanol	HPLC–DAD-MS^n^	Senica et al. (2019)
*Lignans*				
Lignan coumaroyl glucoside	Flowers and leaves	Methanol	LC-DAD-ESI-MS^n^	Qazimi et al. (2024)
*Cyanogenic glycosides*				
Sambunigrin	Berries	Juice, liqueur, tea, and spread	HPLC–DAD-MS^n^	Senica et al. (2016)
*Iridoids*				
Ebuloside	Berries and flowers	Acidified methanol (1% formic acid in methanol)	UHPLC-PDA-MS	Avula et al. (2022)
*p*-Coumaroyl dihydromonotropein	Flowers and leaves	Methanol	LC-DAD-ESI-MS^n^	Qazimi et al. (2024)
*Organic acids*				
Quinic acid	Flowers	50% ethanol and water	LC–MS/MS	Milena et al. (2019)
	Flowers	Aqueous	HPLC–PDA-MS^n^	Ferreira-Santos et al. (2021)
	Flowers and leaves	Methanol	LC-DAD-ESI-MS^n^	Qazimi et al. (2024)
*Fatty acids*				
*α*-Linolenic acid Linoleic acid	Flowers	Methanol	HPLC–DAD-MS	Christensen et al. (2008)
Trihydroxy octadecadienoic acid Trihydroxy octadecenoic acid	Berries and flowers	Acidified methanol (1% formic acid in methanol)	UHPLC-PDA-MS	Avula et al. (2022)
*Triterpenoids*				
Maslinic acid Ursolic acid/Oleanolic acid	Berries and flowers	Acidified methanol (1% formic acid in methanol)	UHPLC-PDA-MS	Avula et al. (2022)

### GC/MS analysis of volatile components and fatty acids in *Sambucus nigra*

Gas Chromatography coupled to Mass Spectrometry (GC/MS) analysis resulted in the identification of hydrocarbons, aldehydes, ketones, alcohols, esters, oxides, and terpenes in addition to some fatty acids in *S. nigra* flowers (Kaack 2008; Kaack et al. 2006; Salvador et al. 2017; Vujanović et al. 2021) and fruits (Hale 2014; Vujanović et al. 2021). Oxygen-containing monoterpenes represented around 98% of the volatile constituents identified in flowers using headspace GC/MS analysis (Salvador et al. 2017), while alkanes were the major constituents of the essential oil obtained from the air dried flowers, accounting for 75% of the identified constituents (Floares et al. 2025). On the other hand, fatty acids and alcohols, triterpenes, and sterols were identified in the berries' dichloromethane extract (Salvador et al. 2015). Additionally, fatty acids were the main components identified in extracts prepared from the leaves and inflorescence, in addition to *n-*alkanes and *n-*alkane esters, triterpenes, sterols, and monoglycerides (Basas-Jaumandreu and de Las Heras 2019). Moreover, (Vujanović et al. 2021) reported constitutional differences in essential oils prepared from air-dried versus lyophilized berries. This means that the composition of volatile, fatty, and other non-polar constituents in *S. nigra* varies according to several factors such as state of the plant material (fresh or dried), drying technique, and whether the analyzed sample is an essential oil or an organic solvent extract. Refer to Table 3 which summarizes the identified components in different *S.* organs through GC/MS analysis.

**Table 3:** Volatile components and fatty acids identified in *Sambucus nigra* (Elderberry) oils and extracts through GC/MS analysis

Name	Organ	Oil/Extract	Identification technique	References
1,8-Cineole 2-Pentylfuran *p-*Cymene Terpinolene 6-Methyl-5-hepten-2-ol *p-*Methoxystyrene β-Ionone	Flowers (frozen)	–	Dynamic head space GC/MS	Kaack et al. (2006)
Eucalyptol Benzaldehyde Methyl benzoate Methyl salicylate	Flowers (frozen)	–	Dynamic head space GC/MS	Kaack (2008)
Pentanal Hexanal Heptanal Octanal Nonanal (*E*,*E*)-2,4-Heptadienal (*E*)-2-Octenal Safranal 1-Penten-3-one 4-Methyl-3-penten-2-one 3-Hydroxy-2-butanone 6-Methyl-5-hepten-2-one 1-Octen-3-one 1-Butanol 2- and 3-Methyl-1-butanol 1-Hexanol (*E*)-2-Hexen-1-ol (*E*)- and (Z)-3-Hexen-1-ol 1-Heptanol 1-Octanol 1-Octen-3-ol *α*-Phellandrene *α* -Terpinene Limonene (*E*)- and (*Z*)-*β*-Ocimene (E)- and (*Z*)-Rose oxide Linalool (*E*)-Linalool oxide Hydroxy linalool Camphor *β*-Caryophyllene *γ*-Terpinene Terpinen-4-ol *α*-Terpineol Hotrienol Citronellol Geraniol Nerol Nerol oxide *β*-Damascenone Benzyl alcohol 2-Phenylethyl alcohol (*Z*)-3-Hexenyl acetate 1,1,6-Trimethyl-1,2- dihydro naphthalene	Flowers (frozen)	–	Dynamic head space GC/MS	Kaack (2008); Kaack et al. (2006)
Phenylacetaldehyde Decane Heptadecane Octadecane Hexanal Isoamyl acetate Limonene Isoamyl alcohol 2-Amylfuran 2-Nonanone Nonanal Furfural Benzaldehyde Linalool 5-Methyl furfural Bornyl acetate Calarene Hotrienol 2-Phenyl ethyl acetate (*E*)-*β*-Damascenone Ethyl dodecanoate Phenyl ethyl alcohol Hexahydro farnesyl acetone 3,4-Dimethyl-5-pentiliden-2-(5H)-furanone 4-Vinyl guaiacol Methyl hexadecanoate Carvacrol Ethyl hexadecanoate Ethyl linoleate Methyl linoleate Methyl linolenate Ethyl linolenate Hexadecanoic acid	Berries (air dried)	Essential oil	GC/MS	Hale (2014)
Decanoic acid Dodecanoic acid Tetradecanoic acid Hexadecanoic acid Octadec-9-enoic acid Octadecanoic acid Eicosanoic acid Tetracosanoic acid Hexacosanoic acid Octadecanol Hexacosanol Campesterol Stigmasterol *β*-Sitosterol *β*-Amyrin Oleanolic acid Ursolic acid	Berries (freeze dried)	Dichloromethane extract	GC/MS	Salvador et al. (2015)
Myrcenol Fenchone Thujone Linalool methyl ether Limonene oxide Tagetone Citronellal Lilac aldehyde Myrtenol Verbenone Lilac alcohol Citral Methyl Citronellate Citronellyl formate Geranial Methyl geranate *α*-Copaene *β*-Bourbonene *β*-Elemene *α*-Bergamotene *β*-Caryophyllene Aromadendrene *α*-Humulene Germacrene D *α*-Farnesene *σ*-Cadinene Calamenen	Flowers (freeze dried)	–	HS-SPME/GC × GC-ToFMS	Sánchez-Hernández et al. (2023)
*n-*Nonadecane *n-*Henicosane *n-*Tricosane *n-*Pentacosane *n-*Docosanol *n-*Tricosanol Methyl tetracosanoate Methyl hexacosanoate Benzyl tetracosanoate Benzyl hexacosanoate Docosanoic acid Hexacosanoic acid 22-Hydroxy cosanoic acid Squalene Monolein L-Isoleucine methyl ester	Inflorescence (fresh)	Pentane:dichloro-methane 7:3 (v/v)	GC-EIMS	Basas-Jaumandreu and de Las Heras (2019)
Levulinic acid Phosphoric acid Tri isobutyl phosphate Phytadiene I Phytol Stearic acid Monopalmitolein *n-*Tetracosanal *n-*Hexacosanal *n-*Henitriacontane *n-*Hexacosanol *n-*Octacosanol	Leaves (fresh)			
*n-*Nonacosane Palmitic acid Linolenic acid *α*-Tocopherol *β*-Sitosterol Campesterol Stigmasterol *α*- and *β*-Amyrin Oleanolic acid Ursolic acid Monopalmitin Monoliolenin Monolinolein	Leaves and inflorescence (fresh)			
*β*-Damascenone (E)-Ocimene *p-*Cymene α- and β-Ionene *Cis*- and *Trans*-Rose oxide *β*-Cyclocitral *α*-Terpineol Ethyl caprylate Isopentyl acetate Linalyl anthranilate Methyl hydrocinnamate 2-Pentyl furan 2,5,5,8a-Tetramethyl-3,4,4a,5,6,8a- hexahydro-2H-chromene Indane-4-carboxaldehyde 5-Methyl-2-phenyl-2-hexenal 2-Hexenol 4-Heptyn-3-ol	Berries (air dried)	Essential oil	GC/MS	Vujanović et al. (2021)
*β*-Damascenone Limonene *Cis*- and *Trans*-* β*-Ocimene Terpinolene *α*- Ionene *Cis*- and *Trans*- Rose oxide *Trans*-*p-*Mentha-2,8-dienol *α*- and *β*-Ionone Linalool *α*-Terpineol 2-Pentyl furan Phytol	Berries (lyophilized)			
Carane *α*-Pinene *Cis*- and *Trans*- Rose oxide 1,2-Methyl-1,4-pentadiene Linalool oxide Caryophyllene *α*-Terpinol Epoxy-linalool *α*-Farnesene *β*-cadinene *α*-Limonene diepoxide *β*-Damascenone 6-Methyl-5-nonadiene-2-on *Cis*- Geraniol *Cis*- Geranylacetone *γ*-Elemene *α*-Caryophyllene oxide *Trans*-2-Caren-4-ol *β*-Caryophyllene oxide *α*-Copaen-11-ol *β*-Methyl ionone 3-*p-*Menthen *α*-Hexyl cinnamaldehyde Linalyl anthranilate Methyl salicylate Methyl-2-hydroxy-1,6-dimethyl cyclohexane carboxylate 3,6-Dihydro-4-methyl pyran 1,3-Isopentyl-cyclopentene 1-Benzyl-1,2,3-triazole Benzopyran 3-Penten-2-ol 2-Penty lfuran 4-Pentyn-2-ol 1-Undecyn	Flowers (air dried)			
Octyl 2-methyl propanoate 3,5-Dihydroxy-6-methyl-2,3-dihydropyran-4-one 2-Propyl malonic acid Dimethyl malonic acid	Flowers (shade dried)	Aqueous ammonia olution	GC/MS	Sánchez-Hernández et al. (2023)
1,6-Anhydro-*β*-D-glucopyranose Oleic acid Butanoic acid pentyl ester	Leaves (shade dried)			
3,3,5-Trimethyl-1,4-hexadiene *β*-Linalool 3,7-Dimethyl-1,5,7-octatrien-3-ol Nonanal *Cis*- and *Trans*-Rose oxide Nerol oxide cis- Dihydroedulan II Dihydroedulan I 6-Methyl-5-(1-methyl ethylidene)- 6,8-nonadien-2-one 6,10,14-Trimethyl- 2-pentadecanone Heptadecane 2,6-dimethyl-2,6-Octadiene Octadecane 1-Octadecanol Nonadecane Eicosane (*Z*)-9-TricoseneHeneicosane Docosane 9-Hexacosene Tetracosane Pentacosane Squalen Tetratriacontane	Flowers (air dried)	Essential oil	GC/MS	Floares et al. (2025)
Palmitic acid Linolenic acid Linoleic acid Oleic acid Stearic acid Arachidic acid Behenic acid Lignoceric acid Cerotic acid Montanic acid		Petroleum ether		

### Analysis of metabolites’ contents of *Sambucus nigra* extracts and the effects of different extraction methods

Various studies focused on determination of metabolites’ contents of different organs of *S. nigra*. These included analysis of total phenolic, flavonoid, and anthocyanin contents. In addition, some studies quantified selected metabolites after their chromatographic identification, especially the major phenolic acids, flavonoids, or anthocyanins in the extracts investigated. The sections below discuss the investigated metabolites contents of *S. nigra* extracts, and the effect of extraction methods applied, which can be a guide for optimization of extraction techniques to prepare extracts rich in desired components.

### Analysis of total phenolic, flavonoid, and anthocyanin contents of *Sambucus nigra* extracts

Total phenolic, flavonoid, and anthocyanin contents (TPC, TFC, and TAC, respectively) vary greatly depending on several factors, including plant organ, extracting solvent, extraction temperature, plant collection location, and application of assisted extraction techniques such as ultrasound or microwave-assisted extractions. For example, with applying same extraction method on flowers and berries of *S. nigra*, the flowers extract showed higher TPC and TFC, whereas anthocyanins were only detected in the berries extract. On the other hand, subjecting the aforementioned organs to an in vitro digestion method resulted in an increase in TPC and a decrease in TFC in both extracts, in addition to diminishing of anthocyanins in the case of the berry extract (Ferreira-Santos et al. 2021). Moreover, upon preparing an anthocyanin-rich extract from *S. nigra* through purification of the berries aqueous extract by either membrane filtration or column chromatography, the later showed higher TPC and TAC compared to the membrane filtered extract (Banach et al. 2021). In addition, the effect of solvent and solvent ratio on TPC and TAC of *S. nigra* pomace was noticed. Extraction with water at 1:30 ratio resulted in the highest TPC, whereas the highest TAC was achieved upon extraction with 70% ethanol at 1:20 ratio (Radványi et al. 2013). Similarly, (Uzlasir et al. 2021) investigated the effects of solvent nature, extraction temperature, and extraction time on TPC of *S. nigra* flowers. The results revealed that extraction with water at 100 °C for 30 min achieved the highest TPC among other investigated extraction conditions. Likewise, (Ferreira-Santos et al. 2021) compared aqueous extraction of *S. nigra* flowers at various temperatures and reported that extraction at 90 °C yielded the highest TPC compared to 50 and 70 °C. Another study implied different extraction techniques: maceration, microwave-assisted, or ultrasound-assisted extractions, together with changing solvents between 50% ethanol and water, and showed that microwave-assisted extraction using 50% ethanol afforded the highest TPC and TFC among others (Milena et al. 2019). Finally, TPC, TFC, and TAC of *S. nigra* berry 80% methanol and flower aqueous extracts varied according to the location of plant collection (Gentscheva et al. 2022; Porras-Mija et al. 2020). Literature studies that investigated TPC, TFC, and TAC of different *S. nigra* organs, together with specifications of the utilized extraction technique, solvent, temperature, and other specified conditions are summarized in Table 4.

**Table 4:** Total phenolic, flavonoid, and anthocyanin contents of extracts prepared from *Sambucus nigra* (Elderberry) organs using various extraction techniques

Organ	Extract	Solvent ratio	Extraction technique	TPC	Unit	TFC	Unit	TAC	Unit	References
Pomace	Aqueous	1:10	Maceration and shaking	32.46 ± 2.66	mg GAE/g dry matter	–	–	44.37 ± 9.54	mg TAC/g dry matter	Radványi et al. (2013)
		1:20		63.28 ± 7.20				68.082 ± 9.87		
		1:30		**69.80 ± 4.15**				58.89 ± 1.78		
	50% ethanol	1:10		29.48 ± 5.22				100.03 ± 4.60		
		1:20		33.69 ± 2.17				90.79 ± 5.09		
		1:30		35.65 ± 0.69				87.83 ± 3.31		
	70% ethanol	1:10		30.56 ± 1.51				117.04 ± 3.29		
		1:20		35.30 ± 1.76				**119.60 ± 3.49**		
		1:30		35.82 ± 1.57				118.37 ± 5.90		
	90% ethanol	1:10		22.94 ± 4.34				54.07 ± 7.96		
		1:20		25.35 ± 1.18				52.57 ± 4.83		
		1:30		23.48 ± 2.38				48.76 ± 1.96		
Branches	95% ethanol	1:40	Boiling with solvent	708 ± 22	mg GAE /100g fresh matter	–	–	253 ± 47	mg CGE/100g fresh matter	Silva et al. (2017)
Berries	1% HCl in methanol	1:20	Maceration and shaking	**1191 ± 85**				**813 ± 156**		
Flowers	50% ethanol	1:30	UAE	362.5	mg GAE /g dry extract	110	mg CE/ g dry extract	–	–	Milena et al. (2019)
			MAE	**417.6**		**115**				
			Maceration	330		100				
	Aqueous		UAE	279.1		60				
			MAE	322.6		100				
			Maceration	200		50				
Berries	80% methanol	1:28	Maceration for 20 h at 4 °C	2.5–3.3 (according to location of collection)	mg GAE /g fresh weight	0.34-0.067 (according to location of collection)	mg QE/g fresh weight	0.45–0.99	mg CGE/g fresh weight	Porras-Mija et al. (2020)
Berries	Aqueous (anthocyanin-rich)	–	Purification by membrane separation	20.52 ± 0.53	mg% CE	–	–	15.24 ± 0.39	mg% CGE	Banach et al. (2021)
			Purification by column chromatography	**48.55 ± 1.78**				**34.28 ± 0.78**		
Flowers	Aqueous	1:30	Extraction at 50 °C for 30 min	20.32	gGAE/100 g of flower dry weight	–	–	–	–	Ferreira-Santos et al. (2021)
			70 °C	25.09						
			90 °C	**30.14**						
Berries	Aqueous (after alkaline and acidic hydrolysis)	–	–	13.28 ± 4.34	mg GAE /g extract	114.98 ± 64.14	mg RE/g extract	109.81 ± 22.62	mg CGE / g extract	Przybylska-Balcerek et al. (2021)
Flowers	80% methanol	1:200	Extraction for 5 min	491.70 ± 9.79	mg GAE/L	–	–	–	–	Uzlasir et al. (2021)
			30 min	621.24 ± 9.42						
	80% ethanol		5 min	347.55 ± 13.82						
			30 min	473.27 ± 13.99						
	Aqueous (100 °C)		5 min	1,118.79 ± 35.17						
			30 min	**1,360.91 ± 26.92**						
	Aqueous (85 °C)		5 min	1,078.18 ± 32.44						
			30 min	1,233.64 ± 34.69						
Blossoms	Aqueous	1:10	Extraction for 20 min at 45 °C	29–49 (according to location of collection)	mg GAE/g Dry Biomass	6–18 (according to location of collection)	mg QE/g Dry Biomass	–	–	Gentscheva et al. (2022)
Flowers	Aqueous	1:30	Extraction at 90 °C for 30 min	156.3 ± 7.1	mg GAE/g dry extract	**59.2 ± 3.3**	mg CE/g dry extract	Not detected	mg CGE / g dry extract	Ferreira-Santos et al. (2022)
			Followed by in vitro digestion	**264.8 ± 14.5**		24.3 ± 1.0		Not detected		
Berries			Extraction at 90 °C for 30 min	100.6 ± 4.6		**22.6 ± 2.5**		**7.07 ± 0.79**		
			Followed by in vitro digestion	**157.1 ± 12.2**		17.3 ± 1.0		Not detected		

Values in bold represents the highest TPC, TFC, or TAC  calculated for the same organ using different extraction processes

Extract ratio: the ratio between plant material weight (g) to solvent volume (ml)

*TPC;* Total phenolic content, *TFC;* Total flavonoid content, *TAC;* Total anthocyanin content, *GAE;* Gallic acid equivalents, *QE;* Quercetin equivalents, *CE;* Catechin equivalents, *CGE;* Cyanidin-3-glucoside equivalents, *RE;* Rutin equivalents, *UAE;* Ultrasound-assisted extraction, *MAE;* Microwave-assisted extraction, *EAE;* enzyme-assisted extraction

### Quantitative analysis of selected metabolites of *Sambucus nigra* extracts

In addition to isolation and metabolites identification of *S. nigra*, some reseraches targeted total or specific metabolites quantification for comparative cross organ or extraction conditions analyses and their effects on metabolites contents. This included quantification of individual phenolic acids, namely; hydroxybenzoic, protocatechuic, gentisic, coumaric, vanillic, gallic, syringic, caffeoylquinic, dicaffeoylquinic, ferulic, ellagic, cinnamic, rosmarinic, benzoic, salicylic, sinapic, and caffeic acids, as well as flavonoids, including hesperidin, apigenin, naringenin, quercetin, rutin, kaempferol, luteolin, vitexin, hyperoside, isoquercitrin, isorhamnetin, isorhamnetin 3-*O*-rutinoside, quercetin rutinoside, morin, catechin, and epicatechin, and anthocyanins such as cyanidin-3-sambubioside and cyanidin-3-glucoside. According to (Ferreira-Santos et al. 2022), the most abundant phenolic acid found in *S. nigra* aqueous flower extract was chlorogenic acid (up to 1198 mg/L), while ferulic acid was the most dominant in aqueous berry extract (132 mg/L). Rutin was the major flavonoid in flower extracts and the only quantifiable one in berry extract (1564 and 162 mg/L, respectively). The same study investigated the effect of in vitro digestion on individual phenolic acids and flavonoid contents, as well as TPC and TFC as mentioned previously. Chlorogenic acid, rutin and quercetin contents decreased or even diminished upon digestion in both flower and berry extracts, whereas the contents of naringenin, ferulic, ellagic, cinnamic, and rosmarinic acids increased. (Przybylska-Balcerek et al. 2021) also revealed that chlorogenic acid and rutin were the most abundant phenolic acid and flavonoid in *S. nigra* aqueous berry extract after alkaline and acid hydrolysis (139 and 1105 mg/g dry extract, respectively). Comparing the effect of using different extraction techniques (maceration, ultrasound- and microwave-assisted extractions) on the phenolic acid and flavonoid contents of aqueous and 50% ethanol extracts of *S. nigra* flowers revealed that chlorogenic acid and rutin were the most abundant phenolic acid and flavonoid in the investigated extracts, and that ultrasound-assisted extraction resulted in the highest contents of them (55 and 91 μg/mg extract, respectively) (Milena et al. 2019). Likewise, comparing between maceration, continuous agitation, and ultrasound-, microwave-, and enzyme-assisted extractions of *S. nigra* berries using 60% ethanol revealed that ultrasound-assisted extraction resulted in the highest contents of rutin, chlorogenic and *p-*coumaric acids, whereas enzyme-assisted extraction resulted in the highest contents of gallic acid, in addition to caffeic and syringic acids, which were not detectable in other extraction techniques.

Chlorogenic acid was the most abundant phenolic acid obtained by all extraction techniques except for enzyme-assisted extraction, where gallic acid predominated (Pascariu et al. 2024). (Yesilada et al. 2014) adopted a more comprehensive quantitation study on the 70% methanol extracts of *S. nigra* leaves, flowers, and berries, and assessed the cyanogenic glycosides content, in addition to phenolic acids, flavonoids, and anthocyanins to ultimately determine which organ is the richest in beneficial polyphenols, and the lowest in content of harmful cyanogenic glycosides. In accordance with other results, chlorogenic acid was the most abundant phenolic acid, and leaf extract was the richest (3355 μg/g dry weight) followed by flowers and berries (2528 and 1098 μg/g dry weight, respectively). Rutin was the most abundant flavonoid with and its highest content is in berry extract (3452 μg/g dry weight) compared to flower and leaf extracts (2656 and 2025 μg/g dry weight, respectively). Anthocyanins were present only in berry extract, with the contents of cyanidin-3- sambubioside and cyanidin-3-glucoside as 2593 and 2282 μg/g dry weight, respectively. Upon summation of the contents of the quantified phenolic acids, flavonoids, and anthocyanins, *S. nigra* berry extract contained the highest total content of these beneficial metabolites. On the other hand, the content of harmful cyanogenic glycosides was the highest in leaf extract and the lowest in berry extract, with the most abundant cyanogenic glycoside being sambunigrin (1006, 379, and 22 μg/g dry weight in leaf, flower, and berry extracts, respectively). Hence, *S. nigra* berries could be considered the safest and most beneficial organ compared to leaves and flowers. Other metabolites such as fatty acid, sugars, amino acids, and organic acids were quantified in different organs of *S. nigra* and their contents are listed in Table 5.

**Table 5:** Contents of fatty acids, sugars, organic acids, and amino acids quantified in different *Sambucus nigra* (Elderberry) extracts

Name	Organ	Content					Unit	References
Fatty acids								
		Supercritical CO_2_ SFE	Hexane					
			PLE	UAE	Soxhlet	Maceration		
Myristic acid	Pomace	0.10 ± 0.00	0.08 ± 0.01	0.08 ± 0.01	0.15 ± 0.02	0.04 ± 0.00	g/100 g pomace	(Kitrytė et al. 2020)
Palmitic acid		7.13 ± 0.09	6.95 ± 0.26	6.78 ± 0.22	9.33 ± 0.18	6.63 ± 0.19		
Palmitoleic acid		0.07 ± 0.01	0.06 ± 0.02	0.07 ± 0.00	0.14 ± 0.01	0.11 ± 0.00		
Stearic acid		1.75 ± 0.00	1.68 ± 0.02	1.72 ± 0.01	1.67 ± 0.01	1.73 ± 0.06		
Oleic acid		12.87 ± 0.04	13.20 ± 0.04	13.14 ± 0.05	12.93 ± 0.05	12.57 ± 0.05		
Linoleic acid		42.00 ± 0.05	42.40 ± 0.30	42.35 ± 0.10	41.40 ± 0.13	40.73 ± 0.00		
γ-Linolenic acid		0.10 ± 0.01	ND	ND	0.10 ± 0.00	0.10 ± 0.00		
α-Linolenic acid		34.13 ± 0.09	33.99 ± 0.35	34.61 ± 0.04	32.04 ± 0.06	32.79 ± 0.20		
Arachidic acid		0.16 ± 0.01	ND	ND	0.11 ± 0.00	0.18 ± 0.01		
Hexanoic acid	Flower	9.94					µg/g dried dichloro-methane extract	Ferreira-Santos et al. (2021)
Nonanoic acid		15.48						
Palmitic acid		59.08						
Oleic acid		18.95						
Stearic acid		38.25						
Behenic acid		17.61						
Lignoceric acid		1.66						
*Sugars*								
Fructose	Flowers	11–18					g/Kg flower fresh weight	Porras-Mija et al. (2020)
Glucose		17–22						
Sucrose		0.05–0.31 (according to location of collection)						
Rhamnose	Flower	32.13					µg/g dried dichloro-methane extract	Ferreira-Santos et al. (2021)
Cellobiose		100.73						
Glucose	Berries	4.89 ± 1.44					mg/g aqueous extract	Przybylska-Balcerek et al. (2021)
Fructose		5.91 ± 1.77						
Sucrose	Leaves	0.55 ± 0.05					g/100 g aqueous extract	Gentscheva et al. (2022)
Glucose		3.19 ± 0.02						
Fructose		2.70 ± 0.06						
Sucrose	Blossoms	0.26 ± 0.03						
Glucose		1.50 ± 0.05						
Fructose		0.79 ± 0.05						
*Organic acids*								
Citric acid	Flowers	2.85–5.52					g/Kg flower fresh weight	Porras-Mija et al. (2020)
Malic acid		2.51–3.67						
Quinic acid		0.72–1.16						
Shikimic acid		0.08–0.1 (according to location of collection)						
Citric acid	Berries	1.03 ± 0.14					mg/g aqueous extract	Przybylska-Balcerek et al. (2021)
Malic acid		0.29 ± 0.05						
Shikimic acid		0.14 ± 0.06						
Fumaric acid		0.07 ± 0.03						
*Amino acids*								
Arginine	Blossoms	0.048–0.051					g/100 g aqueous extract	Gentscheva et al. (2022)
Aspartic acid		0.058–0.063						
Valine		0.028–0.030						
Glycine		0.038–0.042						
Glutamine		0.098–0.113						
Isoleucine		0.029–0.030						
Leucine		0.059–0.062						
Methionine		0.009–0.011						
Proline		0.029–0.031						
Serine		0.028–0.032						
Tyrosine		0.048–0.052						
Threonine		0.029–0.031						
Tryptophan		0.009–0.011						
Hydroxyproline		0.009–0.011						
Phenylalanine		0.038–0.041 (according to location of collection)						
Histidine		0.019–0.021						
Cystine		0.019–0.021						

*SFE;* Supercritical fluid extraction, *PLE;* Pressurized liquid extraction, *UAE;* Ultrasound-assisted extraction, *ND;* not detected

## Traditional uses and ethnopharmacology of *Sambucus nigra*

Different parts of *S.* *nigra* are used as food and herbal supplements due to their high value (Sidor and Gramza-Michałowska 2015; Smith et al. 2021). Traditionally, extracts from the stem bark, leaves, flowers, fruits, and roots are utilized to treat bronchitis, cough, upper respiratory infections, and fever. Elderberries have been historically utilized in various preparations, such as herbal tea, syrup, or juice (Mota et al. 2020; Sala et al. 2023). WHO (World Health Organization) considers its fruits as a diaphoretic herb for the treatment of fever and as an expectorant for mild respiratory tract disorders in addition to relieving common cold symptoms (Knudsen and Kaack 2013). Besides, their uses in the treatment of digestive discomfort, skin conditions, and inflammation (Jarić et al. 2007). As elderberry is a native species throughout Europe and western Asia, in traditional European medicine, elderberry leaves have been primarily used externally to treat skin and dermatological conditions. Decoctions and infusions were used for hemorrhoids, abscesses, eye inflammation, toothache, and gingivitis. Infusions had a laxative effect when used internally, and inhalations helped relieve headaches and reduce fever (Skowrońska et al. 2024). In Turkish traditional medicine, elderberry was used to treat hemorrhoids and wounds, and also to relieve rheumatic pain (Süntar et al. 2010). Furthermore, in the Balkans, fruits were recorded in traditional uses as anti-inflammatory and immunomodulatory candidates due to their richness in flavonoids, anthocyanins, phytosterols, triterpenes, and tannins (Petkova-Parlapanska et al. 2025; Vujanović et al. 2021).

Not only berries, but the traditional uses are extended to elder flowers as in different countries, Albania, Algeria, Italy, and Spain, eldre flowers are used to alleviate bronchial disorders, colds, and stomachaches, and are also employed for their antipyretic, diuretic, digestive, and antirheumatic properties (Motti et al. 2022). Topically, flower decoction is used as a skin toner and whitener (Pieroni et al. 2004). In Italy, Leaves are used to heal local abscesses (De Natale and Pollio 2007). In Palestine, they are used for fever, headache, and urinary tract infections (Kaileh et al. 2007).

Regarding the ethnopharmacological uses, studies have shown that the berries and flowers have anti-inflammatory activity through their effect on nitric oxide (NO) production in lipopolysaccharide (LPS)-stimulated RAW 264.7 macrophages and murine dendritic D2SC/I cells (Ho et al. 2017b). Also their effect on the production of human proinflammatory cytokines interleukins (ILs) {IL-1β, IL-6, IL-8} and tumor necrosis factor-*α* (TNF-*α*) and inflammatory cytokine interleukin 10 (IL-10) was confirmed. The effectiveness of elderberry in relieving influenza virus symptoms was confirmed by in vitro and clinical studies against different types of viruses. It showed a significant effect, especially on the post-infection phase through immunomodulatory property (Kinoshita et al. 2012; Torabian et al. 2019). Additionally, the Ultraviolet radiation (UV) protective effect of elderberry fruits, which was tested as an emulsion, was confirmed (Sidor and Gramza-Michałowska 2015). This effect may protect the skin from skin disorders associated with overexposure to UV, such as erythema, burns, immunosuppression, and even skin cancer (Milutinov et al. 2024). A Supplement containing elderberry extracts with cranberry was tested clinically to confirm its significance against urinary tract infections and its associated symptoms (Mehmood et al. 2019). The wound healing activity of elder leaves was examined and their effect was confirmed, through the exerted inhibition of tyrosinase and hyaluronidase enzymes, as well as their antioxidant properties using cell-free methods (Studzińska-Sroka et al. 2024b).

## Modern pharmacological research on *Sambucus nigra*

Several biological activities have been reported for elderberries, which could be attributed to their phenolic compounds. These activities include antioxidant, immunostimulatory, anti-inflammatory, antiallergic, anticancer, antibacterial, and antiviral (Ferreira et al. 2022b; Pascariu and Israel-Roming 2022). In vitro experiments were extensively used in some bioactivity assessments, such as antioxidant and cytotoxicity assays, while in vivo studies were included in others as in anti-inflammatory and antidiabetic bioassays. The modern pharmacological data reported for elderberry’s different parts are summarized in Table 6.

**Table 6:** Pharmacological activities reported for *Sambucus nigra* (Elderberry) different organs

Pharmacological activity	Type	Model evaluation	Mode of action	References
*Antioxidant*	*Sambucus nigra* L fruit extracts	In vitro free radical scavenging activity (DPPH and ABTS) and *β*-carotene bleaching assays	- Antiradical activities against both ABTS and DPPH - Inhibited *β*-carotene/linoleic acid co-oxidation	Duymuş et al. (2014)
	*Sambucus lanceolata* leaves and fruits methanolic extracts	In vitro by ABTS radical cation, DPPH, NO, and superoxide (O_2_^−^) radical scavenging assays	- Scavenging activity towards DPPH•, ABTS•+, NO and O_2_^−^ radicals	Pinto et al. (2017)
	*Sambucus nigra* L. leaves, berries and flowers alcoholic extracts	In vitro using DPPH and *β*-carotene/linoleic acid methods	- Neutralized the activities of free radicals and inhibited the co-oxidation reactions of linoleic acid and *β*-carotene	Dawidowicz et al. (2006)
	*Sambucus nigra* L. aqueous and anthocyanin enriched extract	In vitro using DPPH, NO, and superoxide (O_2_^−^) radical scavenging assays	- Scavenging activity towards, DPPH•, NO and O_2_^−^ radicals	Neves et al. (2019)
		In vitro neuroprotective effects against rotenone-induced toxicity in SH-SY5Y neuroblastoma cells	- ↓ cytotoxicity induced by rotenone - ↑intracellular GSH/GSSG ratio - ↓ ROS generation promoted by rotenone - ↑activities of SOD, GPx and GR enzymes - Improved mitochondrial respiratory complexes	
	A gelatin–sodium caseinate composite film containing elderberry (*Sambucus nigra* L.) extract 0.5% (w/w)	In vitro by ABTS radical cation and DPPH assays	- Scavenging activity towards DPPH and ABTS - Improved the oxygen barrier properties of the film - Enhanced the edible film’s ability for food quality preservation	Choi et al. (2023)
	*Sambucus nigra* L. pomace extract added to apple juice	In vitro, by using FRAP assay	- ↑ antioxidant compounds in apple juice samples - ↑FRAP value - ↓viable, fungi, and mould counts	Furulyás et al. (2024)
	Yogurt Enriched with *Sambucus nigra* L. fruit extract	In vitro antioxidant using DPPH assay	- Higher scavenging activity towards DPPH - ↓viable microbial count	Pascariu et al. (2025)
	*Sambucus nigra* L. leaves, flowers and fruits methanolic extract	In vitro by using DPPH assay	- Scavenging activity towards DPPH	Nurzyńska-Wierdak et al. (2022)
	*Sambucus nigra* L. fruits and several new elderberry inter- specific hybrids at five maturity stages	In vitro by using DPPH, ABTS, FRAP, and ORAC assays	- Scavenging activity towards DPPH, ABTS, FRAP, and ORAC radicals	Imenšek et al. (2021)
	*Sambucus nigra* L. fruits phenolic extract	In vitro by using DPPH, ABTS, and FRAP assays	- Scavenging activity towards DPPH, ABTS, and FRAP	Rodríguez Madrera and Pando Bedriñana (2023)
	Polysaccharides extracted from Elderberry (*Sambucus williamsii* Hance) fruits	- In vitro protective effect of EEP-2 (acidic polysaccharide) against H_2_O_2_-induced oxidative damage in RAW264.7 cells	- EFP-2 inhibited cellular death induced by H_2_O_2_ oxidative stress - EFP-2 reduced the percentage of apoptosis in RAW264.7	Wei et al. (2022)
		- In vivo protective effects of EFP-2 in a zebrafish model of oxidative damage	- ↓ overall ROS level	
	*Sambucus nigra* L. fruit extract	In vitro antioxidant using hypertrophic 3T3-L1 adipocytes	- ↓intracellular ROS generation - ↓ NOX4 mRNA expression - ↑mRNA expression of antioxidant enzymes, like SOD and GPx	Zielińska-Wasielica et al. (2019)
		In vitro anti-obesity using hypertrophic 3T3-L1 adipocytes	- Modulated the leptin and adiponectin gene expression - ↓leptin expression and secretion - ↑adiponectin mRNA expression and protein secretion in treated adipocytes	
		In vitro antidiabetic using mature 3T3-L1 adipocytes sensitive to insulin and adipocytes treated with TNF-α	- Significant *α*-glucosidase inhibition - Stimulated the 2-NBDG uptake - ↑mRNA expression of GLUT-4	
		In vitro anti-inflammatory using LPS-stimulated RAW 264.7 macrophages	- ↓mRNA expression and protein production of TNF-α and IL-6 - ↓ production of inflammatory mediators PGE_2_ and NO via down-regulation of COX-2 and iNOS expression	
	*Sambucus nigra* L. fruit extract	In vitro antioxidant by using ABTS, FRAP, and ORAC assays	- Scavenging activity towards ABTS, FRAP, and ORAC	Porras-Mija et al. (2020)
		In vitro antidiabetic assay	- Significant inhibition of *α-*amylase and *α*-glucosidase	
		In vitro anti-obesity	- Significant inhibition of lipase	
		In vitro antihypertensive	- Significant inhibition of Angiotensin converting enzyme	
	Elderberry and vine tea compound effervescent tablet	In vitro antioxidant by using DPPH, hydroxyl, and ABTS assays	- Scavenging activity towards DPPH, hydroxyl, and ABTS radicals	Sun (2023)
	*Sambucus formosana* whole plant methanol extract and its fractions	In vitro antioxidant by using DPPH assay	- Methanol and ethyl acetate fractions. exhibited significant DPPH scavenging activity	Huang et al. (2019)
		In vitro antiglycation activity	- Inhibited AGEs formation- activity	
	*Sambucus nigra* L. fruit extract	In vitro antioxidant using DPPH, ABTS, FRAP, and CUPRAC assays	- Strong scavenging activity towards FRAP > DPPH > CUPRAC > ABTS	Has et al. (2023)
	*Sambucus nigra* L. lyophilized powder extract	In vitro antimicrobial capacities	- Significant antimicrobial activity against *Staphylococcus aureus*, and *Pseudomonas aeruginosa*	
	14 accessions of *Sambucus canadens*is L. fruits extract	In vitro using DPPH and FRAP assays	- Scavenging activities towards DPPH and FRAP	Özgen et al. (2010)
	*Sambucus nigra* L. ethanolic extract	In vitro antioxidant using DPPH and CUPRAC assays	- Scavenging activities towards DPPH and CUPRAC	Akduman et al. (2023)
		In vitro antiproliferative against Liver hepatocellular carcinoma cell line (HepG2)	- ↓ HepG2 cell viability	
*Cytotoxic*	Elderberry, concentrated elderberry and elder *Sambucus canadensis* flower extracts of two Canadian cultivars, ‘Kent’ and ‘Scotia’	In vitro cytotoxic against glioma (human brain tumor-derived cell line models (U-87 MG, U-138 MG, Hs 683) and brain microvascular endothelial cells (HBMEC) under both normoxic and hypoxic culture conditions	- Inhibited cancer and endothelial cell growth - Triggered cell cycle arrest and apoptotic cell death - Modulated the expression of several cell cycle checkpoint proteins	Lamy et al. (2018)
	Elderberry-AuNPs	In vitro cytotoxic on the prostate (PC-3) and pancreatic (Panc-1) cancer cells	- ↓ proliferation of PC-3 and Panc-1 cell lines	Sibuyi et al. (2021)
	Ethyl acetate and aqueous acetone extracts from elderberries, as well as detected triterpenoids (ursolic and oleanolic acids)	In vitro cytotoxic on human colon adenocarcinoma cell line (LoVo) and human breast cancer cell line (MCF-7) was investigated by sulforhodamine B assay	- ↓ proliferation of human colon adenocarcinoma cell line (LoVo) and human breast cancer cell line (MCF-7)	Gleńsk et al. (2017)
	Phytoestrogen Extracts Isolated from Elder Flower	In vitro assessment of the effect on hormone production and receptor expression of Trophoblast Tumor Cells JEG-3 and BeWo, as well as MCF7 Breast Cancer Cells	- Inhibited estradiol production - Upregulated ERα in JEG-3 cell lines - In MCF7 cells, a significant ERα downregulation and PR upregulation were observed	Schröder et al. (2016)
*Anti-inflammatory*	*Sambucus nigra* L. lyophilized flowers aqueous extract	In vivo carrageenan-induced inflammation	- ↓ neutrophil migration - ↓ TNF, IL-1β and IL-6 levels - ↓ NO_2_^−^, TNF and IL-6 in LPS-stimulated macrophages	Santin et al. (2022)
		In vitro oyster glycogen-recruited peritoneal neutrophils or macrophages (RAW 264.7) stimulated with LPS	- ↓ NO_2_^−^, TNF, IL-1β and IL-6 - ↑IL-10 in LPS-stimulated neutrophils - ↓ L-selectin (CD62L) and β2-integrin (CD18) expressions - ↑ apoptotic neutrophils efferocytosis of by increasing the IL-10 and decreasing the TNF levels	
		Isolated rutin	- ↓ NO_2_^−^, TNF, IL-1β and IL-6 - ↑ IL-10 in LPS-stimulated neutrophils	
	*Sambucus nigra* L. aqueous and ethanolic leaves extract prepared at room temperature and the solvent’s boiling point	- In vitro anti-inflammatory by LPS- LPS-stimulated human neutrophils - In vitro antioxidant by neutrophils stimulated with bacteria-derived products	- Inhibited TNF-*α* and ROS - Inhibited lipoxygenase activity	Skowrońska et al. (2022)
	Silver nanoparticles (AgNPs), using European black elderberry (Sambucus nigra – SN) fruit extracts	In vivo by carrageenan-induced inflammation	- ↓ edema and cytokines levels in the paw tissues	David et al. (2014)
		In vitro on HaCaT cells exposed to UVB radiation	- ↓cytokines production induced by UVB irradiation	
	*Sambucus nigra* L total extract, and its fractions of three elderberry cultivars, ‘Sabugueiro’, ‘Sabugueira’, and ‘Bastardeira’	- In vitro anti-inflammatory using LPS-stimulated RAW 264.7 macrophage cells	- Inhibited nitric oxide release	Ferreira et al. (2022a)
		In vitro antioxidant using *tert*-butyl hydroperoxide (*t*-BOOH)-induced toxicity on HepG2 and Caco-2 cells	- In Caco-2 cells, elderberry extracts prevented GSH depletion, ROS production, abnormal morphological changes, and DNA fragmentation	
	The ethanol crude extracts from elderberry and elderflower, and isolated anthocyanins, procyanidins, flavonoids, phenolic acids, and other metabolites of *Sambucus nigra* L	In vitro using LPS-activated RAW 264.7 macrophages and murine dendritic D2SC/I cells	- Exhibited strong complement fixating activity - Showed strong inhibitory activity on NO production in RAW cells and dendritic cells	Ho et al. (2017b)
	*Sambucus nigra* L fruit and flower extracts	In vivo anti-inflammatory using cotton pellet-induced granuloma test	- ↓ weight of induced granuloma	Seymenska et al. (2024)
		In vivo antinociptive by using the acetic-acid-induced writhing test	- Inhibited abdominal contractions	
	freeze-dried and oven-dried American elderberry (*Sambucus nigra* subsp. *canadensis*) pomace ethanolic extracts	In vitro anti-inflammatory by using LPS or IFN*γ* stimulated bv-2 mouse microglial cells	- Inhibited induced NO production	Simonyi et al. (2015)
		In vitro antioxidant LPS or IFN*γ* stimulated bv-2 mouse microglial cells	- Inhibited induced ROS production	
	*Sambucus nigra* fruit extract	In vitro by using lipopolysaccharide-activated RAW264.7 macrophages	- ↓ expression of major genes of the inflammatory pathway, such as IL-1*β*, IL-6, TNF-*α* and COX-2 - ↓ IL-6, TNF-*α* and prostaglandin E_2_ secretion - ↓ nitric oxide production	Olejnik et al. (2015)
*Immunomodulatory*	Elderberry (*Sambucus nigra*) extracts	In vitro using bone marrow-derived murine dendritic cells stimulated by *Lactobacillus acidophilus*	- ↑ endocytosis in immature dendritic cells - ↑ IFN-β inducing activity of L. acidophilus in dendritic cells - ↑ production of IL-12 - ↑ proinflammatory cytokines IL-6 and TNF-α in dendritic cells - ↑ IL-1β production	Frøkiær et al. (2012)
	*Sambucus ebulus* fruit infusion	200 mL infusion intake in healthy humans	- ↓ pro-inflammatory status a in total protein, IL-6, TNF-*α*, and IL-8 - ↓ complement activity markers C3 and C4	Kiselova-Kaneva et al. (2023)
	*Lactobacillus rhamnosus* CRL1505 with *Sambucus nigra* L. flowers and fruits glyceric extract	- In vitro on human monocytes (THP-1) - ex vivo on human peripheral blood mononuclear cells (PBMC)	- ↑ IL-6 and IL-10 expression in PBMC	Cappellucci et al. (2024)
	13% anthocyanin standardized elderberry (EB) extract	In vitro conditions using spleen and thymus lymphocytes	- ↑ spleen and thymus T-cell proliferation - ↑ spleen B-cell proliferation by EB-extract - ↑IL-2 levels	Khan et al. (2022)
	Elderberry derived water extract (EC15) and its polysaccharide enriched fractions (CPS, BOUND, and UNBOUND)	In vitro effect on Immune Phenotype of Dendritic Cells (DCs)	- Induced DC maturation - ↑ T cell stimulation - ↑ IL-6, TNF-*α*, and IFN-*γ*	Stich et al. (2022)
*Neuroprotective*	A SC-Nanophytosomes formulation based on enriched elderberry anthocyanins extract (EAE) and *Codium* lipids	In vitro assays with Neuroblastoma SH-SY5Y cells using rotenone- and glutamate-induced toxicity	- Improved the mitochondrial respiratory chain complexes I and II, and preserved the mitochondrial membrane potential in the presence of rotenone - Protected the SH-SY5Y cells against the toxicity induced by rotenone or glutamate	Mendes et al. (2021)
		Rat Model of Parkinson’s Disease induced by rotenone	- Reverted the *α*-synuclein levels and antioxidant enzymes activity - Mitigated mitochondrial dysfunction	Mendes et al. (2022)
	Elderberry (2%EB) diet	Cellular ataxia (CA) rat models were generated by 3-acetylpyridine-induced (3-AP) administration	- Improved motor coordination, locomotor activity, and neuro-muscular function - Prevented Purkinje neurons degeneration - ↑ microglia and astrocyte complexity - ↓ cell soma size - ↓ apoptosis in the cerebellum of 3-AP ataxic model - ↓ expression of inflammatory, apoptotic, and necroptotic genes - ↑ expression of antioxidant-related genes	Siasi et al. (2024)
		Rat model of irritable bowel syndrome	- Improved locomotion and decreased anxiety-like behavior in the rat models of IBS - ↓ TNF-*α* expression - ↑ mucosal layer thickness and the number of goblet and mast cells in colon tissue samples - Prevented astrogliosis and astrocyte reactivity	Namakin et al. (2023)
		Rat Models of Alzheimer’s Disease Induced by Amyloid Beta Injection	- Improved memory and learning function - Prevented the degeneration of hippocampal neurons	Jahanbakhshi et al. (2022)
		In vivo using Huntington’s disease (HD) rat model	- Recovered motor failure and muscle incoordination - ↓ 3-NP induced growth in caspase-3 and TNF-*α* concentration - Improved striatal antioxidative capacity by a significant reduction in ROS and a remarkable rise in GSH	Moghaddam et al. (2021)
	Elderberry (2%EB) diet	Methamphetamine-induced toxicity in rats	- Improvement in the methamphetamine-induced memory decay and anxiety-like behaviors - Recovered the population spike (PS) amplitude and the field excitatory postsynaptic potential (fEPSP) slope of the evoked potentials of the LTP components in hippocampus neurocircuits - ↓ change in the astrocyte morphology and prevents the astrocyte reactivity in the hippocampus of rats following the methamphetamine administration - Improved neuronal cell arrangement in the hippocampus of rats	Khanjari et al. (2025)
	Elderberry extract (2%EB)	Protection against intrahippocampal A*β*-induced memory dysfunction in rats	- Improved the memory functions of rats with Aβ toxicity - ↓ astrogliosis and astrocytes process length and the number of branches and intersections distal to the soma - ↓ caspase-3 expression in the hippocampus of rats with A*β* toxicity - Protected hippocampal pyramidal neurons against A*β* toxicity and improved the spatial distribution of the hippocampal neurons - ↓ expression of inflammatory and apoptotic genes	Jahanbakhshi et al. (2023)
		Tramadol-induced toxicity in the hippocampus of adult male rats	- Improved most of the memory-related indices in tramadol - fEPSP slope and PS amplitude returned to control levels - ↓ caspase-3 expression in hippocampal cells - ↓ astrogliosis	Sohrabi et al. (2025)
	American elderberry (*Sambucus nigra* subsp. canadensis) juice	Effect on cognition and inflammatory markers in patients with mild cognitive impairment (MCI)	- ↓ several markers of low-grade peripheral inflammation, including vasorin, prenylcysteine oxidase 1, and complement Factor D	Curtis et al. (2024)
*Antimicrobial and Immunomodulatory*	*Sambucus nigra* fruit extracts	In vitro antiviral activity against H1N1 infection (human influenza A) using Madin-Darby canine kidney (MDCK Cells)	- Bounded to H1N1 virions, blocking host cell entry and/or recognition	Roschek et al. (2009)
	*Sambucus nigra juice*	In vitro antiviral activity against MDCK cells using hemagglutination inhibition assay and plaque reduction assay	- Blocked the viral glycoproteins, HA and NA, in the early and late stages of the influenza infection cycle - ↓ number of plaques and size of some of the plaques, demonstrating inhibition of infection propagation	Torabian et al. (2019)
		In vitro immunomodulatory using fluorescent microbead array probing	- ↑ production of IL-6, IL-8, and TNF	
	*Sambucus nigra* standardized liquid extract	In vitro antiviral and antibacterial activity using the microtiter broth micro-dilution assay	- Antimicrobial activity against both Gram-positive bacteria of *Streptococcus pyogenes* and group C and G Streptococci - Antimicrobial activity against Gram-negative bacterium *Branhamella catarrhalis* - Inhibited the propagation of human pathogenic influenza viruses H5N1-type influenza A virus isolated from a patient and an influenza B virus	Krawitz et al. (2011)
	Anthocyanin-enriched elderberry (*Sambucus nigra)* fruit extract (eldosamb®)	In vitro antiviral activity	- Antiviral efficacy against the MVA virus (modified vaccinia virus Ankara)	Schön et al. (2021)
		Ex vivo immunomodulatory activity	- ↓ secretion of pro-inflammatory cytokines IFN-*γ* and TNF-*α* - ↑ IL-4 and IL-10 - ↓ IL-2 - Modulated the Th1/Th2 response with a shift towards the Th2-Helper cell response	
	*Sambucus nigra* fruit extract and extract dried powder	In vitro antiviral activity against SARS-CoV-2	- Inhibited the replication of SARS-CoV-2 and the production of progeny virions - Inhibited replication of SARS variant Alpha, Beta, Gamma, Delta and Omicron	Setz et al. (2023)
	*S. nigra* flowers and leaves methanolic extracts	In vitro anti-dengue serotype-2 activity	- Both extracts exhibited anti-DENV-2 activity, particularly when DENV-2 was pre-incubated with the extracts before being added to cell cultures	Castillo-Maldonado et al. (2017)
	Antiviral activity of *Sambucus Formosana* Nakai stems ethanol extract	In vitro against human coronavirus NL63 (HCoV-NL63)	- ↓ cytopathicity and virus yield in HCoV-NL63-infected cells	Weng et al. (2019)
*Cardiovascular protective*	Polyphenol-rich black elderberry extract (BEE)	In vitro on intestinal cholesterol metabolism using Caco-2 Cells	- ↓ mRNA and protein levels of genes for cholesterol absorption, such as Niemann–Pick C1 Like 1 and ATP-binding cassette transporter A1 (ABCA1) - Induced low-density lipoprotein receptor, ABCG5/G8, and ABCB1 - ↓ expression of genes for lipogenesis and altered the mRNA levels of sirtuins - Stimulated the transintestinal cholesterol excretion (TICE)	Jeon et al. (2021a)
	Polyphenol-rich black elderberry extract	In vivo using an L-NAME-induced experimental model of arterial hypertension (AHT)	- A combination of a renin inhibitor (Aliskiren) and polyphenolic extract generated a superior antioxidant effect compared to administering the two separately - ↓ TAS and TAD in rats with drug-induced hypertension	Ciocoiu et al. (2016)
	Anthocyanin-rich black elderberry extract (*Sambucus nigra*) (BEE) (13% anthocyanins)	In vivo against inflammation-related impairments in HDL function and atherosclerosis in apoE^−^/^−^ mice	- ↓ aspartate transaminase (AST) and fasting glucose - Improved HDL function (Apoa1, Pon1, Lcat, Clu) - ↓ hepatic cholesterol levels (increased Ldlr and Hmgcr, reduced Cyp7a1) - ↑ serum paraoxonase-1 (PON1) arylesterase activity - ↓ serum chemokine (C–C motif) ligand 2 (CCL2) - ↓ total cholesterol content of the aorta, indicating less atherosclerosis progression	Farrell et al. (2015a)
	Lyophilized pomace of elderberry obtained extracts MeW, DCM and HEX extracts	In vitro Endothelial Nitric Oxide Synthase (eNOS) Activity using [^14^C]-L-arginine to [^14^C]-L-citrulline conversion assay in the human endothelium-derived cell line EA.hy926	- Enhanced the A23187- - stimulated eNOS activity	Waldbauer et al. (2018)
	Elderberry extracts (EB)	In vitro assessment of endothelial dysfunction (ED) markers improvement using Human umbilical vein endothelial cells (HUVEC)	- Prevented TNF-*α* induced apoptosis and reactive oxygen species production in HUVECs - ↑ Akt and eNOS activity, and Nrf2 expression in response to TNF-*α* - ↓ NOX-4 expression and NF-κB activity - Prevented the adhesion of monocytes to HUVECs - ↓ IL-6 and MCP-1 levels, which was associated with inhibition of VCAM-1 expression	Festa et al. (2023)
	Polyphenol-rich black elderberry (BEE)	Protective effects of BEE on oxidative stress and hepatic cholesterol metabolism cells using an in vitro assay and HepG2 cells	- Scavenging activity towards, DPPH, and ABTS radicals - ↓ sterol regulatory element-binding protein 2, 3-hydroxy-3-methylglutaryl coenzyme A reductase, and low-density lipoprotein receptor - Marked induction of genes for high-density lipoprotein metabolism, i.e., scavenger receptor class B type 1and ATP-binding cassette (ABC) transporter A1 - ↑ expression of canalicular efflux transporter for hepatic cholesterol and bile acids, such as ABCG5/G8 and ABCB11 - ↓ expression of genes for fatty acid oxidation - Altered the expression of histone deacetylase and sirtuins	Jeon et al. (2021b)
*Antidiabetic*	Elderberry (*Sambucus nigra* L.) cultivars flower extracts	In vitro enzyme inhibition targeting enzymes critical in carbohydrate digestion and glucose regulation	- Black Beauty, Obelisk, and Haschberg cultivars demonstrated significant inhibition of α-glucosidase, with a high inhibitory potential against α-amylase enzymes for the Obelisk cultivar	Studzińska-Sroka et al. (2024a)
		In vitro antioxidant using DPPH scavenging and CUPRAC methods	- Significant antioxidant for DPPH and CUPRAC radicals	
		- In vitro anti-inflammatory using hyaluronidase inhibition and LPS-stimulated RAW 267.4 murine macrophage model	- Inhibited hyaluronidase enzyme - Inhibited of NO release	
	Elderberry extracts and its isolated Anthocyanin, procyanidin, and its metabolites	In vitro enzyme inhibition using substrate oxidation, *α*-amylase and *α*-glucosidase	- Enhanced glucose and oleic acid uptake by skeletal muscle - Inhibited *α*-amylase and *α*-glucosidase enzymes	Ho et al. (2017a)
		In vitro antioxidant using DPPH, 15-LO (15- lipoxygenase) and XO (xanthine oxidase) assays	- Significant radical scavenging activity towards DPPH - Significant inhibition of 15-LO and XO	
	Elderberry (*Sambucus nigra* L.) lipophilic and polar extract	In vivo using STZ-Induced Diabetic Rats Fed with a High-Fat Diet	- The polar extract modulated glucose metabolism by correcting hyperglycemia - lipophilic extract lowered insulin secretion - Both extracts lowered insulin resistance	Salvador et al. (2016)
	*Sambucus nigra* L. rich polyphenolic extract	In vivo using STZ-Induced Diabetic Rats	- ↓ glycosylated hemoglobin values - ↓ lipids peroxides, neutralize the lipid peroxide radicals and inhibit the LDL oxidation - Preserved the atherogenic risk is at normal limits - ↑ serum activity of glutathione-peroxidase and superoxide-dismutase	Ciocoiu et al. (2009)
	Elderberry fruit extract (lipophilic (LFSn) and phenolic (PhFSn) fractions)	Evaluation of Calcium and Magnesium Status in STZ-Induced Diabetic Rats	- Both extracts normalized the kidney Ca content - PhFSn extract decreased the liver Mg content in diabetic rats	Krejpcio et al. (2024)
*Anti-obesity*	Anthocyanin-dense elderberry juice (EBJ)	Meal tolerance test (MTT)	- Increased Firmicutes and Actinobacteria, and decreased Bacteroidetes at the phylum level - Increased *Faecalibacterium*, *Ruminococcaceae*, and *Bifidobacterium* and decreased *Bacteroides* and lactic acid-producing bacteria at the genus level - ↓ blood glucose following the MTT - ↑ fat oxidation during the MTT and 30 min of moderate physical activity with the EBJ treatment	Teets et al. (2024)
	Elderberry (*Sambucus nigra* L.) juice powder	Diet-induced obesity in animal models	- ↓ body weight gain, fat pads, and whole-body fat - Improved fasting blood insulin despite eating significantly more kilocalories and maintaining the same physical activity - Increased *Bifidobacterium*, and promoted *Akkermansia* and *Anaeroplasma* - Prevented *Desulfovibrio* growth	Minj et al. (2024)
	Anthocyanin-rich black elderberry extract (BEE)	Diet-induced obese C57BL/6 J mouse model	- ↓ lower liver weights, serum triglyceride (TAG), homoeostasis model assessment and serum monocyte chemoattractant protein-1 - ↓ serum insulin and TNF*α* - ↓ hepatic fatty acid synthase mRNA - ↓ PPARγ2 mRNA and liver cholesterol suggesting decreased hepatic lipid synthesis - ↑ adipose PPAR*γ* mRNA, transforming growth factor *β* mRNA and adipose tissue histology suggested a pro-fibrogenic phenotype that was less inflammatory in 1·25%-BEE treated group - ↑ skeletal muscle mRNA expression of the myokine IL-6	Farrell et al. (2015b)
	Whole blackberries (BBs)	Volunteers consuming a high-fat diet using meal-based glucose tolerance test (MTT)	- ↓ average 24 h respiratory quotient (RQ) - ↑ fat oxidation - Improved insulin sensitivity	Solverson et al. (2018)
*Anti-infertility*	Elderberry (*Sambucus nigra L*.) extract (EB) and extract-derived monosaccharide-amino acid (FL)	H_2_O_2_-induced decrease in testosterone-deficiency syndrome in a TM3 Leydig cell	- ↓ H_2_O_2_-induced intracellular ROS levels - Improved testosterone secretion - ↑ mRNA and protein expression levels of steroidogenesis-related enzymes (StAR, 3*β*-HSD, 17*β*-HSD, CYP11A1, CYp17A1) - Inhibited the conversion of testosterone to estradiol by elderberry extract and extract-derived FL, which reduced the mRNA and protein expression of CYP19A1	Lee et al. (2024)
	Elderberry diet (2%EB)	Transient Scrotal Hyperthermia‐Induced Mice	- Improved sperm parameters and stereological parameters, like spermatogonia, primary spermatocyte, round spermatid, and Leydig cells together with an increasing level of the serum testosterone - ↓ expression of TNF-*α* and caspase-3	Moghaddam et al. (2022)
	Elderflower and elderberry extracts	In vitro assessment of secretion activity of steroid hormones 17*β*-estradiol and progesterone by Immortalized human ovarian granulosa cell line (HGL5 cells)	- ↑ 17*β*-estradiol and progesterone release	Baldovska et al. (2021)

## Safety and interactions of *Sambucus nigra*

Elder berries and flowers are considered safe food supplements by the US Food and Drug Administration (FDA) and the European Medicines Agency (EMA) (Fossum 2014). This safety was confirmed in a recent study (Seymenska et al. 2024) with results showing that the lethal doses (LD_50_) for the dry fruit extract ranged from 3 to 5 g/kg body weight, while for the dry flower extract, it ranged from 300 to 500 mg/kg body weight, depending on the route of administration.

Although elder flowers and berries are considered safe, they may cause nausea, vomiting, and abdominal cramps with high doses (Tsui et al. 2001), and some precautions were reported regarding the long-term use of berries (Ulbricht et al. 2014). For example, elderberry may have a lowering effect on blood pressure, and thus caution is required for patients taking blood pressure medications (Hasani-Ranjbar et al. 2009). Also, elderberry may cause tachycardia, raising a red flag for patients with arrhythmia or cardiac disorders. This effect is due to its content of cyanogenic glycosides, which are converted to hydrogen cyanide during digestion, leading to cyanide poisoning (Ulbricht et al. 2014). Some reports indicated possible interaction(s) of *S. nigra* with chemotherapy medications e.g., a case study reported for interaction with chemotherapy used in sarcoma (panzopanib) (Agarwal and Mangla 2024). Similar cautions should be considered for diabetic patients due to its effect on glucose metabolism and insulin release (Gray et al. 2000), and for concurrent use of diuretic medications, as elderberries may have diuretic action in high doses (Beaux et al. 1999). (iii).

Of note, animal nor clinical studies regarding the safety of *S. nigra* during pregnancy were found. Therefore, it is recommended for healthcare professionals not to prescribe elderberry to treat upper respiratory tract infections during pregnancy or lactation (Holst et al. 2014).

## Conclusion

Plants have a wide range of bioactive compounds that have a substantial impact on consumers’ health. *Sambucus nigra* L. (elderberry) is traditionally used as a medicinal plant by many native peoples and herbalists. This review aimed to shed light on the different traditional and research-based biological activities of elderberry and its secondary metabolites. Moreover, as far as natural product chemists, nutritionists and health care providers are concerned about optimized activity and/or metabolite(s) enrichment, this review also covered the literature for effects of different extraction conditions applied to elderberry and their impacts on the chemical composition, and hence the biological profile of resulted extracts. Finally, the analytical tools employed to study elderberry have been summarized to be used as references and guidelines for any future studies. Stem bark, leaves, flowers, fruits, and root extracts are used to treat bronchitis, cough, upper respiratory infections, and fever as listed in several pharmacopoeias, recognized textbooks, and monographs. Numerous research-based studies have recommended that consuming elderberry preparations has positive health effects, as it has the potential to treat different respiratory problems and infections, colds, cardiovascular disorders, diabetes, and obesity. Furthermore, considerable enhancement of the immune system, in addition to antimicrobial activities, and UV radiation protection were also reported. Chemically, elderberry is a good source of several important and valuable secondary metabolites, particularly phenolic compounds, including phenolic acids, flavonoids, and anthocyanins. LC/MS and GC/MS analyses were mainly used to study, characterize, and monitor chemical discrepancies of elderberry extracts and preparations. Regarding optimized extracts preparation, different organs, collection locations, and extraction conditions (e.g., solvent(s) and extraction temperatures) were covered to optimize the phenolic content and minimize cyanogenic glycosides content. Despite the beneficial findings, more research is still needed to understand how interactions with other molecules/drugs may alter the activity profile and potency of elderberry components. So far, literature sources have not supplied solutions to the issues concerning the interaction mechanism(s) of elderberry components, their stability during storage, or their use as a safe functional food.

## Data Availability

No datasets were generated or analysed during the current study.

## Abbreviations

ABTS: 2,2′-Azinobis-(3-ethylbenzthiazoline-6-sulfonic acid)
AGEs: Strong advanced glycation end-products
AuNPs: Gold nanoparticles
CD62L: L-selectin
Clu: Clusterin/apolipoprotein J
COX-2: Cyclo-oxygenase-2
CUPRAC: Cupric ion reducing antioxidant capacity
Cyp7a1: Cholesterol 7α-hydroxylase
DPPH: 2,2-Diphenyl-1-picrylhydrazyl
ERα: Estrogen receptor alpha
fEPSP: Field excitatory postsynaptic potential
FRAP: Ferric reducing ability power
GLUT-4: Glucose transporter type 4
GSH: Reduced glutathione
GSSG: Oxidized glutathione
GPx: Glutathione peroxidase
GR: Glutathione reductase
Hmgcr: HMG-CoA reductase
3β-HSD: 3β-Hydroxysteroid dehydrogenase
H_2_O_2_: Hydrogen peroxide
IL: Interleukin
iNOS: Inducible nitric oxide synthase
IFNγ: Interferon-γ
LCAT: Lecithin-cholesterol acyltransferase
L-NAME: *N*(G)-nitro-_L_-arginine-methyl ester
LPS: Lipopolysaccharide
NOX-4: NADPH oxidase isoform 4
2-NBDG: 2-*N*-7-(Nitrobenz-2-oxa-1,3-diazol-4-yl) amino-2-deoxy-_D_-glucose)
NO: Nitric oxide
NO_2_^−^: Nitrite
Nrf2: Nuclear factor erythroid 2-related factor 2
3-NP: 3-Nitropropionic acid
ORAC: Oxygen radical absorption capacity
PON1: Paraoxonase-1
PGE_2_: Prostaglandin E2
PR: Progesterone receptor
PS: Population spike
ROS: Reactive oxygen species
SARS-CoV-2: Severe Acute Respiratory Syndrome Coronavirus 2
SAA1: Serum amyloid
SOD: Superoxide dismutase
STZ: Streptozotocin
StAR: Steroidogenic acute regulatory protein
TAD: Diastolic blood pressure
TAS: Systolic blood pressure
TNF-α: Tumor necrotic factor-alpha
